# Associations of maternal age at marriage and pregnancy with infant undernutrition: Evidence from first‐time mothers in rural lowland Nepal

**DOI:** 10.1002/ajpa.24560

**Published:** 2022-05-24

**Authors:** Jonathan C. K. Wells, Akanksha A. Marphatia, Mario Cortina‐Borja, Dharma S. Manandhar, Alice M. Reid, Naomi M. Saville

**Affiliations:** ^1^ Population, Policy and Practice Research and Teaching Department UCL Great Ormond Street Institute of Child Health London UK; ^2^ Department of Geography University of Cambridge Cambridge UK; ^3^ Mother and Infant Research Activities Kathmandu Nepal; ^4^ UCL Institute for Global Health London UK

**Keywords:** adolescent pregnancy, child marriage, infant and child undernutrition, rural lowland Nepal, stunting

## Abstract

**Objectives:**

Maternal factors shape the risk of infant undernutrition, however the contributions of age at marriage versus age at pregnancy are rarely disentangled. We explore these issues in a population from lowland rural Nepal, where median ages at marriage and first pregnancy are 15 and 17 years respectively and marriage almost always precedes pregnancy.

**Methods:**

We analyzed data on first‐time mothers (*n =* 3002) from a cluster‐randomized trial (2012–2015). Exposures were ages at marriage and pregnancy, categorized into groups. Outcomes were z‐scores for weight (WAZ), length (LAZ), head circumference (HCAZ), and weight‐for‐length (WLZ), and prevalence of wasting and stunting, for neonates (<8 days) and infants (6–12 months). Mixed linear and logistic regression models tested associations of marriage and pregnancy ages with outcomes, adjusting for parental education, household assets, caste, landholding, seasonality, child sex, intervention arm, randomization strata and cluster.

**Results:**

For neonates, pregnancy <18 years predicted lower LAZ, and <19 years predicted lower WAZ and HCAZ. Results were largely null for marriage age, however early pregnancy and marriage at 10–13 years independently predicted neonatal stunting. For infants, earlier pregnancy was associated with lower LAZ and HCAZ, with a trend to lower WAZ for marriage 10–13 years. Early pregnancy, but not early marriage, predicted infant stunting.

**Conclusions:**

Early marriage and pregnancy were associated with poorer growth, mainly in terms of LAZ and HCAZ. Associations were stronger for neonatal than infant outcomes, suggesting pregnancy is more susceptible to these stresses. Early marriage and pregnancy may index different social and biological factors predicting child undernutrition.

## Graphical abstract

- Early marriage and early pregnancy showed independent associations with the risk of infant undernutrition in a low‐income rural setting in lowland Nepal.
- Early marriage acted primarily as a gateway to early reproduction, whereas early pregnancy was associated with infant undernutrition both at birth and in the second 6 months.
- The main associations of early pregnancy with infant outcomes were deficits in linear growth markers, suggesting that infants recover from earlier deficits in nutritional status by re‐allocating energy from linear growth.

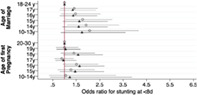

## 1 INTRODUCTION

Child undernutrition in low‐ and middle‐income countries is associated with a range of socio‐ecological stresses, including poverty, food insecurity, exposure to infectious diseases and other markers of social inequality. For three decades, research in this context has been guided by a conceptual model developed by UNICEF (UNICEF, 1990). According to this framework, “immediate causes” of undernutrition include inadequate dietary intake and high infection rates; “underlying causes” include insufficient access to food, inadequate health infrastructure, poor care and feeding practices, while “basic causes” include the lack of financial and socio‐economic resources available to households (e.g., education and employment) and inadequate political will (UNICEF, 1990).

The majority of research drawing on this framework has addressed the “immediate” and “underlying” causes, and in this context, the importance of constraints on the mother is already well recognized (Black et al., 2013; Victora et al., 2008; Victora et al., 2021). Various markers of maternal undernutrition, including short stature, low body mass index (BMI), micronutrient deficiency and inadequate dietary intake have all been linked with adverse effects on growth and nutritional status of the next generation (Addo et al., 2013; Griffiths et al., 2016; Motamed et al., 2019; Ozaltin et al., 2010; Rahman et al., 2015; Rao et al., 2001). Broader biological models of development identify the primary environmental influence experienced by the fetus and breast‐fed infant during early critical windows of development as maternal phenotype (Wells, 2010), a term that encompasses any quantifiable maternal trait in the broad sense. From an evolutionary perspective, maternal phenotype may be assessed in terms of diverse components of “maternal capital,” which refers to any characteristic that promotes maternal investment in the offspring (Wells, 2010). In terms of the immediate and underlying causes of undernutrition, relevant components of maternal capital include maternal body size and nutritional status, which shape the physiological capacity to transfer nutrients to the offspring. According to this approach, low energy stores and micronutrient deficiencies indicate depleted maternal capital, reducing the capacity for maternal nutritional investment.

Less attention has been directed towards the “basic” causes of child undernutrition embedded in broader society (Harris & Nisbett, 2020), though here again the importance of maternal phenotype has been recognized. Three decades ago, UNICEF had already recognized both the subordination of women as “a very important community‐level political and basic economic cause” of child undernutrition, and that “the power structure both within and among households is often legitimized by traditional ideologies, which are often imbedded in the accepted local culture” (UNICEF, 1990). Consistent with that approach, empirical research has found that maternal education and empowerment may benefit child nutrition through their impact on factors such as women's control of their time, and household income and resources, and on their mental health, confidence, and self‐esteem (Bhagowalia et al., 2012; Smith et al., 2003). In the opposite direction, studies have linked maternal exposure to intimate partner violence with an increased risk of child undernutrition (Rahman et al., 2012; Tsedal et al., 2020; Ziaei et al., 2014). Here again the concept of maternal capital is relevant, as education, autonomy, wealth, and supportive social networks can all be conceptualized as forms of social/human capital, that contribute to overall maternal capital by promoting maternal nutritional investment in the offspring, including through receiving help and support from others (Wells, 2010).

Societal factors may therefore affect infant nutrition by promoting or depleting maternal capital. The composite effects of societal gender inequality for child outcomes were demonstrated by an analysis across 96 countries, showing that the “Gender Inequality Index”, a composite index constructed from data measuring women's disadvantage relative to men, was significantly associated with rates of low birth weight, and stunting, wasting and mortality in children below 5 years (Marphatia et al., 2016). Consistent with these associations, a review by Smith and Haddad (2015) found that improvements in women's education and empowerment and gender equality are among the key drivers of past reductions in stunting.

One of the ways in which society profoundly shapes maternal capital is through the scheduling of marriage. According to the UN, marriage before age 18 years represents a fundamental violation of human rights (UN General Assembly, 2014), yet in many countries it remains common for women's marriage to take place before this threshold (UNICEF, 2014, 2021). In Nepal, for example, where our study was undertaken, despite a legal minimum age of 20 years, or 18 years with parental consent, 33% of women aged 20–24 years married <18 years in 2019, of which 8% were married <15 years (UNICEF, 2021). In both South Asia and East Africa, early marriage of women has been identified as a strong risk factor for stunting among children in the age range birth to 5 years (Efevbera et al., 2017; Khan et al., 2019; Paul et al., 2019; Prakash et al., 2011; Raj et al., 2010). However, there are several underlying pathways that may contribute to this association.

First, early marriage can be considered a “cultural gateway” for early reproduction, since pregnancy outside marriage is rare in this society. Early marriage and pregnancy directly impact growth and nutritional status of offspring, especially of first‐borns. Studies in South Asia in particular have linked early marriage with lower age at first birth and higher fertility, which reduces the inter‐birth interval (Raj et al., 2009; Santhya et al., 2010). In turn, several studies have associated adolescent reproduction with increased rates of low birth weight (Alves et al., 2013; Fall et al., 2015; Thirukumar et al., 2020). Among the underlying mechanisms may be competition between mother and offspring for nutrients, which may depress the growth of either mother (Marphatia et al., 2021b) or fetus (Scholl et al., 1994), and reduced dimensions of the obstetric pelvis (Alves et al., 2013). However, the literature reports inconsistent associations of early marriage with maternal nutritional status. For example, while a study from India associated early marriage with a higher prevalence of low maternal BMI, categorized as <18.5 kg/m^2^ (Prakash et al., 2011), a study of 103 DHS surveys from 35 African countries found that after adjusting for early childbearing, early marriage itself was associated with a reduced risk of maternal undernutrition (Efevbera et al., 2019).

Second, early marriage may adversely affect child growth by undermining maternal social and human capital. For example, early marriage is associated with reduced maternal education (Marphatia et al., 2020), and may also contribute to psychosocial stress due to the low position of such women in the marital household hierarchy (Ahmed et al., 2013). This psychosocial stress may then impact child growth by impairing both placental nutrition and lactation through physiological mechanisms, through the action of hormones such as cortisol (Baker et al., 2018; Dejin‐Karlsson et al., 2000; Mohd Shukri et al., 2019). In the same population, for example, we recently showed that early marriage, independent of age at pregnancy, is associated with an increased risk of delivering a baby preterm (Miller et al., 2021). Early marriage has also been associated with shorter maternal height, independent of early reproduction (Marphatia et al., 2021b). Reduced maternal autonomy, such as lack of access, or the opportunity to take children for medical care, has similarly been linked to child undernutrition (Doan, & Bisharat, L., 1990; Shroff et al., 2008). Notably in this population, wives tend to eat only after other household members have done so, potentially reducing both the quantity and quality of their nutritional intake (Harris‐Fry et al., 2018; Morrison et al., 2018), and younger wives may be at a particular disadvantage in this context due to their more subordinate status in the household.

The relative importance of these biosocial pathways may differ across settings. In the population where our study is based in the Nepal Terai, levels of maternal education are very low, and even where women attain education, it rarely translates into the opportunity to earn income outside the family home. In our study sample, over three quarters of women had not attended any school at all, and only those who had stayed in secondary school until at least the 9th grade were likely to marry above the age of 18 years (Marphatia et al., 2020). Natal families tend to pay a dowry when their daughters marry. As the cost of dowry increases with a girl's age and education level, poorer families may be discouraged from sending their daughters to school, contributing to the combination of lack of education and early marriage.

In many populations, child marriage defined as marriage before 18 years (UNICEF, 2014) represents a relatively rare event, and the aim of research is to compare the outcomes of such girls against the majority reference group. In our study population, conversely, the median age at marriage is 15 years, and only ~15% were married after the minimum legal age of 18 years (Marphatia et al., 2020). In prior analyses of this cohort, we found that education is the primary predictor of delayed marriage (Marphatia et al., 2020), and conversely that low natal household wealth is not a direct predictor of early marriage but does predict lack of schooling for girls (Marphatia et al., 2021a). However, caste, land ownership, household assets, household location and girls' education in combination explained less than a quarter of the risk of early marriage (Marphatia et al., 2021).

Although we have limited ability to explain the variability in individual girls' and women's marriage age, however, our study allows us to examine how child undernutrition is associated with variability in age at marriage and pregnancy in a population where chronic undernutrition and lack of schooling are widespread (Marphatia et al., 2020; Ministry of Health, Nepal, 2017), the entire population across all castes is exposed to powerful norms of gender inequality, and girls may be married from very early adolescence onwards. We focus on first‐time mothers, as the psychological consequences of early marriage may be greatest among such women, and the shorter duration between exposures (marriage, pregnancy) and outcomes makes the detection of such associations both more feasible, and more relevant for understanding the health impacts of early marriage on children.

**FIGURE 1:**
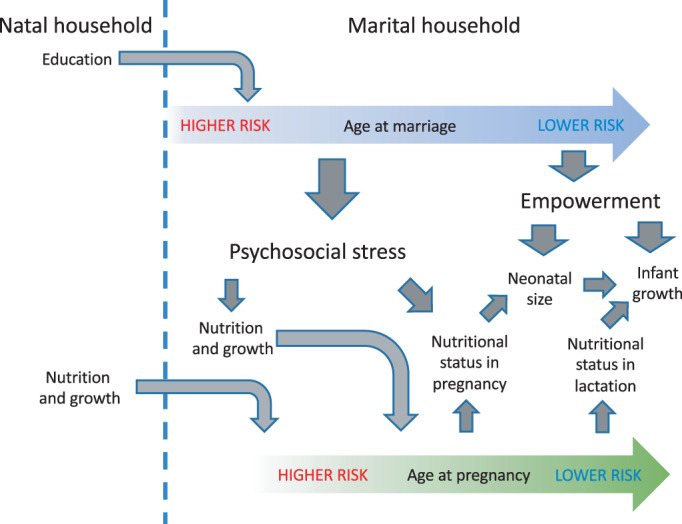
Conceptual diagram for the analysis. We hypothesize that early age at marriage might impair fetal and infant growth through its association with elevated psychosocial stress and reduced empowerment, due to a new wife having low position in the family hierarchy in the marital household. We hypothesized that early age at pregnancy might be associated with smaller maternal body size and with lower nutrient stores (energy, micronutrients), thus constraining the ability of the mother to nourish the fetus via placental nutrition and the infant via lactation. Both earlier marriage and earlier pregnancy are therefore hypothesized to confer high risk of infant undernutrition, with this risk decreasing in dose–response manner for marriage or pregnancy at later ages.

We hypothesize that earlier categories of girls' and women's age at marriage and first pregnancy are independently associated with poorer child nutritional status, and that this will be detected in our samples measured at birth and in the second half of infancy (6–12 months). Our conceptual framework is illustrated in Figure 1, and highlights that we assume that the pathways linking early marriage with child undernutrition (psychosocial stress, reduced empowerment) are in large part different from those that link early pregnancy (anatomical and metabolic constraints). We also discuss the language used to describe girls and women in this population, in the context of early marriage, in Box 1.

BOX 1 **Child marriage and early marriage: finding language to explore the issues.**

In 1963, the Nepal Government defined the minimal acceptable age at marriage for both sexes as 18 years (His Majesty's Government of Nepal, 1963). Recently, the minimal age for marriage was increased to 20 years (Government of Nepal, 2017). Anyone married below this age is defined as having experienced a child marriage.

These legal mandates were drawn up because early marriage prevents individuals from gaining the full benefits of childhood, a stage of development that is supposed to be free of adult responsibilities (Melchiore, 2013). Those married as children have missed out on the opportunity to gain the skills, education, empowerment necessary to achieve economic and social success in adult life. Moreover, girls married as children have had insufficient time to gain the nutrient reserves required to nourish their offspring during pregnancy and lactation, and to gain the autonomy needed for their maternal role.

In the Madhesi population in the lowland Terai region, however, marriage before 18 years remains the norm and around 85% of our sample were married before this age (Marphatia et al., 2020). This creates challenges in relation to the language used to describe our study participants. If we use the term “child” to describe someone who is married, living in the marital household and already a mother by the age of 16 years, we ignore how she has acquired multiple responsibilities of adult women during her adolescent years. If we use the term “woman,” however, we ignore the lost years of childhood. It should also be noted that when it is the norm for girls to be married early in adolescence, there may be social penalties for delaying marriage, leading to psychosocial distress for those remaining unmarried.

Regardless of their age at first pregnancy, which occurs before 19 years in 60% of our sample, by the very nature of our study design all participants are already both wives and mothers. They have had to take on two key roles that define adult life. We have therefore used the phrase “girls and women” but highlight that in this population the boundary between these terms is clouded by the norm of early marriage.

This results in a paradox in that we are studying how infant growth in the next generation relates to being born to both (a) mothers who are themselves still children, and (b) girls who have experienced an accelerated transition to the role of women.

## 2 METHODS

Our study focuses on the predominately Maithili‐speaking Madhesi population, living across Dhanusha and Mahottari districts in Province 2 of the lowland Terai region of Nepal, a plains area which lies to the south of the Himalayan mountain range. The data come from the cluster randomized controlled “Low Birth Weight South Asia Trial” (LBWSAT), conducted between 2013 and 2015 (Saville et al., 2016; Saville et al., 2018); Trial registration: ISRCTN75964374. This assessed the impact of pregnancy interventions on birth weight and weight‐for‐age z‐score in children aged 0–16 months. The trial protocol and main results are described elsewhere (Saville et al., 2016; Saville et al., 2018; Saville et al., 2020; Style et al., 2017). Briefly, all married women aged 10–49 years residing in 80 Village Development Committees (VDC) in the two selected districts who had not had operative family planning, and whose husbands had not had vasectomy were eligible to participate in the trial. A total of 64,000 eligible women consented to menstrual monitoring, and 25,090 pregnancies among 24,682 women were recruited into the trial (Saville et al., 2018). VDC clusters were randomized to one of four arms: (a) Participatory Learning and Action (PLA) women's groups behavior change approach to improve nutrition and care for pregnant women, (b) PLA with unconditional cash transfers, (c) PLA with a fortified blended food supplement, or (d) control group where usual Government of Nepal health services were available.

Research ethics approval for the original trial was obtained from the Nepal Health Research Council (108/2012) and University College London (UCL) Research Ethics Committee (4198/001). Consent for inclusion of villages in the trial was obtained from VDC officials. Written consent was obtained from women regardless of their age with guardians also consenting to participation of married adolescents (<18 years of age). Additional ethical approval for the secondary analysis was obtained from UCL (0326/015), the University of Cambridge Department of Geography (1016), and the Nepal Health Research Council (292/2018).

### 2.1 Data collection

Questionnaires were administered orally in the Maithili language to pregnant women by trained fieldworkers, using smartphones. Data were collected on current age, age at marriage and first pregnancy, education level of the woman and her husband, caste, parity, and indicators of household wealth. Women's age at marriage and when they first became pregnant were recorded in “running years” and converted to completed years by subtracting 1 year. Data collection from each participant continued through the trial and was scheduled to cover early and/or late pregnancy, delivery, and early infancy. At the trial's endpoint, when children ranged in age from birth to 22 months, a cross‐sectional endpoint study captured information on maternal and child anthropometry.

A household wealth score was derived using principal component analysis. Each household was assigned a score based on the ownership of 11 items, including ownership of consumer goods (television, motorbike, and computer), and household infrastructure, including source of drinking water, toilet facilities, materials for flooring, roof and walls, number of rooms, access to electricity and non‐biomass fuel use. The first principal component had positive factor loadings for all 11 variables and accounted for 31.2% of the variability, compared to 10.9% and 9.7% from the second and third principal components respectively. The first score was thus taken as the marker of wealth.

The 11 contributing the highest factor loadings to the first principal component listed in decreasing order (weight shown in parenthesis) were: wall (0.393), toilet (0.382), roof (0.382), flooring (0.371), motorbike (0.311), number of rooms used for sleeping (0.302), television (0.272), access to electricity (0.237), drinking water source (0.209), non‐biomass cooking fuel use (0.174) and computer (0.157). Land‐holding was excluded from the asset score because we wanted to test its association with having a girl, independent of material wealth.

Maternal height and infant length were measured using Shorr boards (precision 1 mm). Infant weight was measured using Tanita BD590 scales (precision 10 g), and head circumference with Seca 212 tapes. Anthropometric z‐scores were calculated using WHO 2006 growth standards and cleaning criteria (Crowe et al., 2014).

### 2.2 Sample selection

In order to be able to interrogate associations of age at marriage and age at pregnancy with infant anthropometry without confounding by parity or a large gap in time between marriage and pregnancy, we restricted the analysis to first‐time mothers. Due to our study design, maternal age at pregnancy acts as a strong proxy for maternal age. A flow chart showing the stages of sample selection is given in Figure S1. Of the 408 women with more than one pregnancy in the study, the first pregnancy was selected for analysis, yielding a potential sample of 24,682 mothers. Of these, 8544 (34.6%) were first‐time mothers (primigravida) and were eligible for inclusion in the analysis. None had missing data for age of first pregnancy. After excluding multiparous women, we excluded women who had married before 10 years (*n =* 34) or after 24 years (*n =* 23), and also women whose first pregnancy was after 30 years (*n =* 57) as these were outliers in the population. This left a total of 8430 mothers with data on age at marriage and/or first pregnancy, of whom 3002 had children who were measured before 8 days and/or between 180 and 365 days. These 3002 children provided 1388 anthropometric datapoints for neonates and 2289 datapoints for infants 180–365 days (infant endpoint measurements were made at varying ages), as described in more detail below.

### 2.3 Data processing

We categorized women's age at pregnancy into the following groups: 10–14 years, 15, 16, 17, 18, 19, and 20–30 years. We categorized women's age at marriage into the following groups: 10–13 years, 14, 15, 16, 17, and 18–24 years.

Educational attainment, recorded as the highest grade in school respondents studied to in school, was categorized into 3 groups for the woman and her husband according to the structure of the education system in Nepal and the low prevalence of education above secondary level: never went to school, primary/lower secondary (classes 1–8); secondary and above (class 9 up to bachelors or higher) (Ministry of Education Nepal, 2016). Caste affiliation was categorized as disadvantaged (Dalit, Muslim), middle (Janjati, other Terai), or relatively advantaged (Yadav, Brahmin). The wealth score data were converted into tertiles. Land ownership was treated as binary.

Infant anthropometry was measured prospectively at three time points (birth, after 42 days, and cross‐sectionally at trial endpoint when children ranged from 0 to 22 months in age). Since ages at both prospective and cross‐sectional measurements were very variable, we treated the data as a mixed longitudinal sample whereby we divided the data into two periods for analysis. First, we categorized anthropometric measurements made before 8 days from birth as neonatal data, indicating size at birth and providing an index of nutritional status at the end of maternal nutritional investment during pregnancy. Second, we categorized anthropometric measurements undertaken during the period 180–365 days after birth as infant data, providing an index of nutritional status after the end of the period when maternal nutritional investment was primarily via breast‐feeding. Absolute values for length, weight, weight‐for‐length, and head circumference were converted into age‐ and sex‐specific z‐scores using WHO reference values, and termed LAZ, WAZ, WLZ, and HCAZ respectively.

### 2.4 Statistical analysis

To describe the associations between age at marriage and age at pregnancy in the sample, we generated a heat map which displayed the frequency of women in each combination of marriage age and pregnancy age.

We initially ran minimally‐adjusted linear regression models (Model 1) with neonatal and infant anthropometric z‐scores as outcomes, and either age at marriage or age at pregnancy as the predictor. We entered categorical terms for each marriage or pregnancy age group (defined above) as appropriate, using the highest values (marriage 18+ years; pregnancy 20+ years) as the reference group. All models adjusted for randomization cluster as a random effect and for age in days for infant (but not neonatal) outcomes. This was because population level growth faltering occurs in cumulative linear manner through the first 2 years of life.

Additional models (Models 2 and 3) then adjusted for potential confounders or mediating factors (maternal and paternal education; wealth tertile; caste; land ownership; study arm; randomization strata; and child sex). As previous analyses of this cohort have demonstrated associations of both sex and seasonality with neonatal and infant anthropometry (Saville et al., 2022a; Saville et al., 2022b), we adjusted for sex and season. Season of measurement was adjusted for by entering the terms “cosinor” harmonic terms in the models, where day of the year is quantified numerically along the scale 1–365 and 1–182.5, for annual and half‐yearly seasonality respectively. We derived “cosinor” harmonic terms for the day of measurement as follows:

$$\text{sine cosinor term}=\sin\left(\frac{\mathrm{Y\pi t}}{D}\right)$$

$$\text{cosine cosinor term}=\cos\left(\frac{\mathrm{Y\pi t}}{D}\right)$$

where sin(.) and cos(.) are trigonometric functions of time *t* (in days out of the period of 365 or 182.5), with π=3.14159 *, D* = 365 or *D* = 182.5 *(*the total number of days in a year or half‐year for annual and half‐yearly seasonality, respectively), and *Y* = 2 for annual and *Y* = 4 for half yearly terms. Then pairs of sine and cosine terms for annual and half‐yearly seasonality were added to the adjusted models to ensure that season of measurement was fully adjusted for.

We then entered both marriage age and pregnancy age together in the same minimally‐ and fully‐ adjusted regression models, to test whether the associations were independent of one another. In exploratory analyses, we also assessed the effects on these models of adding in data on maternal height, which were only available on subsamples (Model 3). These exploratory analyses were intended to give an indication of whether maternal size or long‐term nutritional status contributed to associations of age at marriage or pregnancy with offspring outcomes, to stimulate future research.

### 2.5 Sensitivity analysis

Finally, we conducted a sensitivity analysis, testing the association of early pregnancy with offspring growth among those who had also married early. Despite having analyzed marriage age and pregnancy age in combination, as described above, this additional analysis was warranted because, since marriage almost universally precedes reproduction in this population, there were no women in our sample with the combination of early pregnancy and late marriage. We therefore investigated the association of age at pregnancy with outcomes among those who had married at or below 15 years (median marriage age in this population), adjusting also for age at marriage. We analyzed the same categories of pregnancy age as described above, and also marriage at 10–13 years, relative to those marrying at 14 or 15 years (reference category in these analyses).

All models were run in Stata 15.1 (StataCorp LLC, College Station, TX). In keeping with recent recommendations (Amrhein et al., 2019), we focus on reporting magnitudes of association and their 95% confidence intervals, however we also provide all numerical outputs including *p*‐values in supplementary tables.

## 3 RESULTS

A description of the 3002 women who satisfied the inclusion criteria and whose offspring had at least one anthropometric data‐point is given in Table 1. Of these, 220 had missing data for marriage age, making a slightly reduced sample size for the statistical models investigating marriage age, or the two exposures in combination. The majority of both fathers and mothers had never attended school, while one third belonged to either Dalit or Muslim socially disadvantaged groups. However, around two thirds of the households owned land. A total of 14% of the mothers had short stature <145 cm. Inter‐relationships between caste, education, and indices of wealth are given in Table S1. Dalit or Muslim households had lower levels of education, household assets and land ownership, and higher prevalence of maternal short stature.

**TABLE 1:** Respondent characteristics (*n* = 3002)

Characteristics	*n =* 3002	
	Freq.	%
Age at marriage (Refs. 18–24)	*n =* 2782	
10–13 years	146	5.2%
14 years	438	15.7%
15 years	678	24.4%
16 years	618	22.2%
17 years	498	17.9%
18–24 years	404	14.5%
Age 1st pregnancy (Refs. 20–30)	*n =* 3002	
10–14 years	42	1.4%
15 years	232	7.7%
16 years	444	14.8%
17 years	808	26.9%
18 years	369	12.3%
19 years	491	16.4%
20–30 years	616	20.5%
Mother education	*n =* 3000	
Never went to school	1489	49.6%
Primary to lower secondary	828	27.6%
Secondary and above	683	22.8%
Husband education	*n =* 3000	
Never went to school	1261	42.0%
Primary to lower secondary	859	28.6%
Secondary and above	880	29.3%
Asset tertile (11 assets, no land)	*n =* 2982	
Poorest	858	28.8%
Middle	1014	34.0%
Least poor	1110	37.2%
Caste	*n =* 3002	
Dalit/Muslim disadvantaged	1002	33.4%
Janjati/Other Terai castes middle	1341	44.7%
Yadav/Brahmin least disadvantaged	659	22.0%
Owns land	*n =* 2998	
No	980	32.7%
Yes	2018	67.3%
Randomization stratum	*n =* 3002	
Small, inaccessible	683	22.8%
Small, accessible	682	22.7%
Large, inaccessible	776	25.8%
Large, accessible	861	28.7%
Study arm woman enrolled in	*n =* 3002	
Control	695	23.2%
Women's group	663	22.1%
Cash	788	26.2%
Food	856	28.5%
Sex of infant	*n =* 3002	
Boy	1567	52.2%
Girl	1435	47.8%
Mother's height	*n =* 2310	
<145 cm	319	13.8%
145–147.9 cm	362	15.7%
> = 148 cm	1629	70.5%

Table S2 compares primigravid women with multigravid women in the LBWSAT cohort, and also sampled primigravidae with primigravidae for whom child measurements were not available. As they belong to a younger “generation” of women within the overall cohort, primigravidae represent a subgroup likely to be wealthier and more educated. Compared with multigravidae, they differ in terms of age of marriage and pregnancy, education of the woman and her husband, caste, land ownership, maternal height, and sex of the child. Compared to those lacking data, our sample was slightly less educated, had more middle Terai castes, were more likely to come from small inaccessible clusters, and less likely to have been in the cash transfer study arm. Although these differences are statistically significant, the differences amount to only 1–4 percentage points, so we view our sample to be broadly representative of younger first‐time mothers and their children in Dhanusha and Mahottari districts.

**FIGURE 2:**
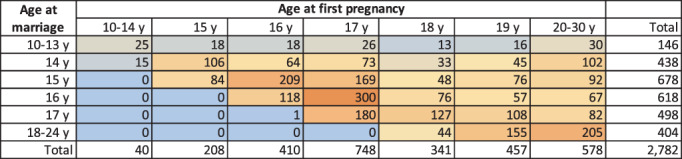
Heat map of age at first pregnancy by age at marriage in first‐time mothers. Frequencies range from low values (blue shading) to high values (orange shading).

Figure 2 provides a heat map looking at the frequency of age at first pregnancy by marriage age among 2782 primigravid mothers who had both age at marriage and first pregnancy data. Women who married very early had very variable age at first pregnancy, whereas by 16 years pregnancy tended to occur the year after marriage. The most common experience was of marriage at 16 years and first pregnancy at 17 years. Among those who married at 17 years, the most common outcome was that they also became pregnant at 17 years. Note that the final row in the table has a wider range for age at marriage and does not necessarily imply that women marrying at 18+ years waited much longer to get pregnant.

Associations of age at marriage and age at pregnancy with socioeconomic variables are given in Tables S3 and S4, respectively. Earlier age at marriage was associated with lower education of both husband and wife, lower household assets and land ownership, greater prevalence of maternal short stature, and greater proportion of Dalit or Muslim caste. Earlier age at pregnancy showed similar associations but the patterns were much weaker (Table S6). However, marriage before 18 years was the norm in every caste grouping, with a prevalence of 82% in Yadav/Brahmin castes, 85% in Janjati or other Terai castes, and 88% in Daly/Muslim castes (Table S5). Likewise, at least 59% of girls of all castes were pregnant by 18 years.

The 3002 children provided up to 1387 neonatal (<8 days) anthropometric datapoints, depending on the specific outcome, and 2281 infant (180–365 days) datapoints, with 666 (~22%) measured during both timepoints. Nearly all children were measured once within each of the sampling periods, with the exception of 7 infants who were measured twice during the 180–365 day period. Average values for anthropometric z‐scores are shown for neonates and infants in Table 2. All mean z‐scores were well below 0, ranging from −1.64 to −0.86, indicating high levels of underweight, linear growth faltering and wasting in this population. As expected, average z‐scores were lower in infancy compared to the neonatal period, indicating continued growth faltering through the post‐natal period. For example, the prevalence of stunting increased from 24.7% at birth to 31.1% in infancy.

**TABLE 2:** Neonatal and infant anthropometry and malnutrition in the whole sample of children of first‐time mothers.

	Anthropometric z‐scores		
Neonates (first week <8 days)	*n*	Mean	*SD*
LAZ	1364	−1.34	1.11
WAZ	1387	−1.40	1.02
WLZ	1192	−0.86	1.24
HCAZ	1364	−0.96	1.17
Infants (second 6 months 180–365 days)	*n*	Mean	*SD*
LAZ	2281	−1.50	1.13
WAZ	2262	−1.64	1.06
WLZ	2252	−1.02	1.00
HCAZ	2277	−1.52	1.01

	Undernutrition		
Neonates (first week <8 days)	*n*	Frequency	%
Stunting	1364	337	24.7
Wasting	1192	204	17.1
Infants (second 6 months 180–365 days)	*n*	Frequency	%
Stunting	2281	710	31.1
Wasting	2252	344	15.3

*Note*: Stunting defined as LAZ < –2 z‐scores; Wasting defined as WLZ < –2 z‐scores. Only 666 infants (~2% of the sample of 3002) were measured at both time points.

Abbreviations: HCAZ, head circumference z‐score; LAZ, length z‐score; WAZ, weight z‐score; WLZ, weight‐for‐length z‐score.

### 3.1 Neonatal analyses

A graphical example of the minimally‐adjusted Model 1 and adjusted Model 2 for neonatal LAZ and age at marriage and pregnancy is given in Figure S2, however for clarity all figures presented in the main article provide only results for models 1 and 2 for the two exposures (marriage and pregnancy age), with full numeric results for these exposures given in supplementary tables. In the example in Figure S2, education of the father, advantaged caste and female infant sex were associated with higher LAZ at birth, but not maternal education, wealth, caste, land ownership, season, randomization strata or study arm. Results for model 3, adjusted for maternal height as well as other covariates, are also provided in Tables S2–S9. Results for covariates can be seen in Figure S3, with greater maternal education associated with greater neonatal WAZ, WLZ, and HCAZ, advantaged caste associated with greater WAZ and HCAZ, and greater wealth associated with greater WAZ.

When analyzed separately, age at marriage showed no association with neonatal LAZ, WAZ, WLZ, and HCAZ. Although there was a trend to lower LAZ and HCAZ among those marrying younger, the confidence intervals overlapped zero for all age groups. These patterns were very similar across models 1 and 2 (Table S6). Conversely, earlier age at pregnancy was associated with lower neonatal LAZ, WAZ, WLZ, and HCAZ, relative to the reference group of age at first pregnancy between 20 and 30 years (Table S7). Broadly, in each case there was an indication of a dose–response association whereby the earlier the age at pregnancy, the larger the anthropometric deficit, with effect size ranging from −0.19 to −0.55 z‐scores across the different outcomes. However, the confidence intervals were much larger for the earliest age at pregnancy (10–14 years) due to the smaller sample size, and only for HCAZ did they not span zero.

**FIGURE 3:**
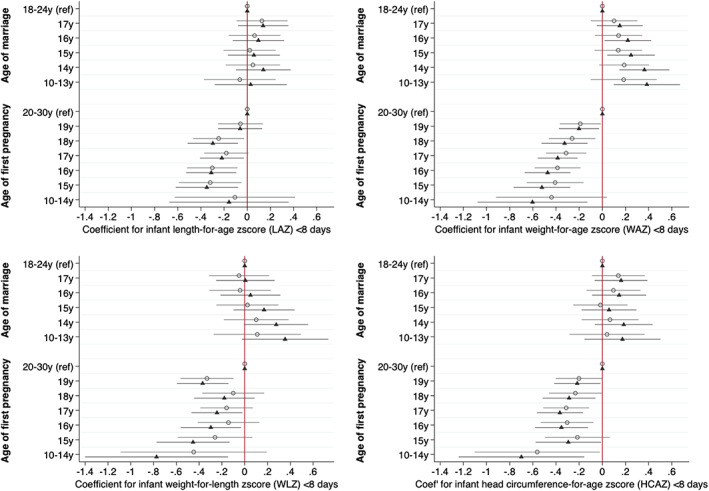
Coefficients from minimally and fully adjusted models of neonatal anthropometric z‐scores by age at marriage and pregnancy (mutually adjusted) for length‐, weight‐ and head circumference‐for‐age, and weight‐ for‐length z scores in the first 8 days of life. Open circles represent coefficients from models adjusted for clustering using random effects only. Shaded triangles represent models additionally adjusted for maternal and paternal education, asset tertiles, caste, land ownership, seasonality (cosinor terms) randomization strata, study arm and child sex as fixed effects. Reference categories: Age at marriage 18–24 years; age at first pregnancy 20–30 years (numeric values given in Table S4).

Figure 3 (numeric values in Table S8) shows the models analyzing neonatal outcomes in relation to age at marriage and at pregnancy in combination. Earlier pregnancy was associated with lower LAZ from 18 years and below, and lower WAZ and HCAZ from 19 years and below. Associations for WLZ were significant after adjustment at 19 years, 16, 15 and 10–14 years. The magnitudes of reduction in LAZ and HCAZ associated with early pregnancy ranged from −0.22 to −0.36 LAZ and −0.20 to −0.68 HCAZ, though confidence intervals remained wide for the earliest age‐group (≤14 years). Independently, associations with marriage were not significant for LAZ and HCAZ, while WAZ was actually greater (coefficients ranging from 0.22 to 0.39) in adjusted models for those marrying at or below 16 years, and a trend in the same direction was apparent for WLZ for those marrying at or below 14 years (coefficients 0.28 and 0.36). Adjusting for maternal height attenuated the association of early pregnancy with LAZ but made little difference to the other associations.

**FIGURE 4:**
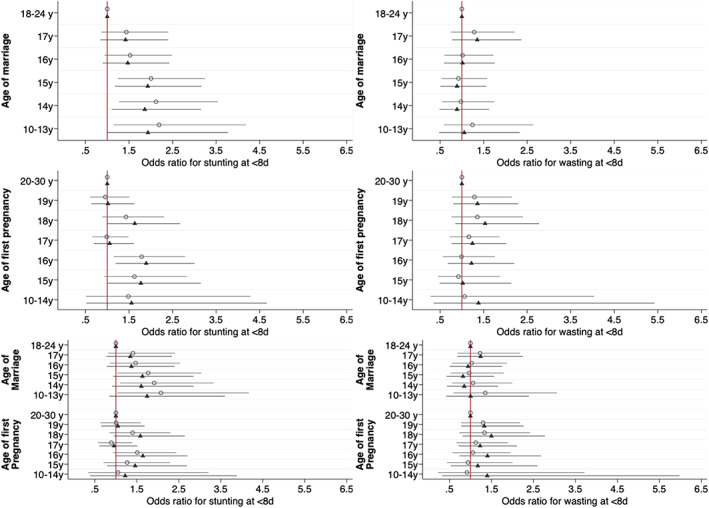
Odds ratios from minimally and fully adjusted logistic regression models of neonatal stunting and wasting in the first 8 days of life against age at marriage and age at first pregnancy separately and mutually adjusted. Open circles represent coefficients from models adjusted for clustering using random effects only. Shaded triangles represent models additionally adjusted for maternal and paternal education, asset tertiles, caste, land ownership, seasonality (cosinor terms) randomization strata, study arm and child sex as fixed effects. Reference categories: Age at marriage 18–24 years; age at first pregnancy 20–30 years (numeric values given in Table S8).

Figure 4 shows equivalent plots for the odds of neonatal stunting and wasting (numeric values in Table S9, coefficients for covariates illustrated in Figure S4). Analyzed separately, both early marriage and early pregnancy were associated with an increased risk of neonatal stunting but not wasting, with the effects evident among those marrying at 15 years or younger or being pregnant at 16 years or younger. In the combined minimally adjusted models, risk of stunting was elevated in those marrying at or below 15 years, whereas associations were weak and not significant for pregnancy age. In combined adjusted models, marriage age association were slightly reduced in magnitude, but the overall pattern remained. Risk of stunting was higher in those marrying at 15 years (adjusted OR 1.63, 95% CI 0.94, 2.85), 14 years (adjusted OR 1.61, 95% CI 0.91, 2.85) and 10–13 years (adjusted OR 1.74, 95% CI 0.84, 3.60). The risk was also elevated among those pregnant at 18 years (OR 1.58, 95% CI 0.95, 2.64) and 16 years (OR 1.64, 95% CI 1.00, 2.71), but the pattern showed less consistency across age groups. Adjusting for maternal height, the risk of stunting remained elevated in those marrying below 17 years (OR ranging from 1.89, 95% CI 0.97, 3.69, for those marrying at 16 years; 2.29, 95% CI 0.98, 5.41, for those marrying 10–14 years. The results for neonatal wasting were all null.

### 3.2 Infant analyses

Overall, outcomes in the second half of infancy were less strongly associated with age at either marriage or pregnancy. When analyzed separately, early age at marriage at 10–13 years was associated with lower infant LAZ (adjusted ∆ = –0.30, 95% CI –0.54, −0.06), however for other categories of early marriage the coefficients were much smaller (Table S10). There was no association of marriage age with the other outcomes. Earlier pregnancy was associated with lower infant LAZ, with the deficit increasing from ~0.2 z‐scores for pregnancy 15 to 18 years to –0.38 z‐scores for 10–14 years (Table S11). For WAZ and HCAZ, associations were largely null, except for pregnancy age 10–14 years where adjusted models showed larger deficits (HCAZ: adjusted ∆ = −0.34, 95% CI –0.71, −0.03; WAZ: adjusted ∆ = −0.35, 95%CI –0.74, 0.03).

**FIGURE 5:**
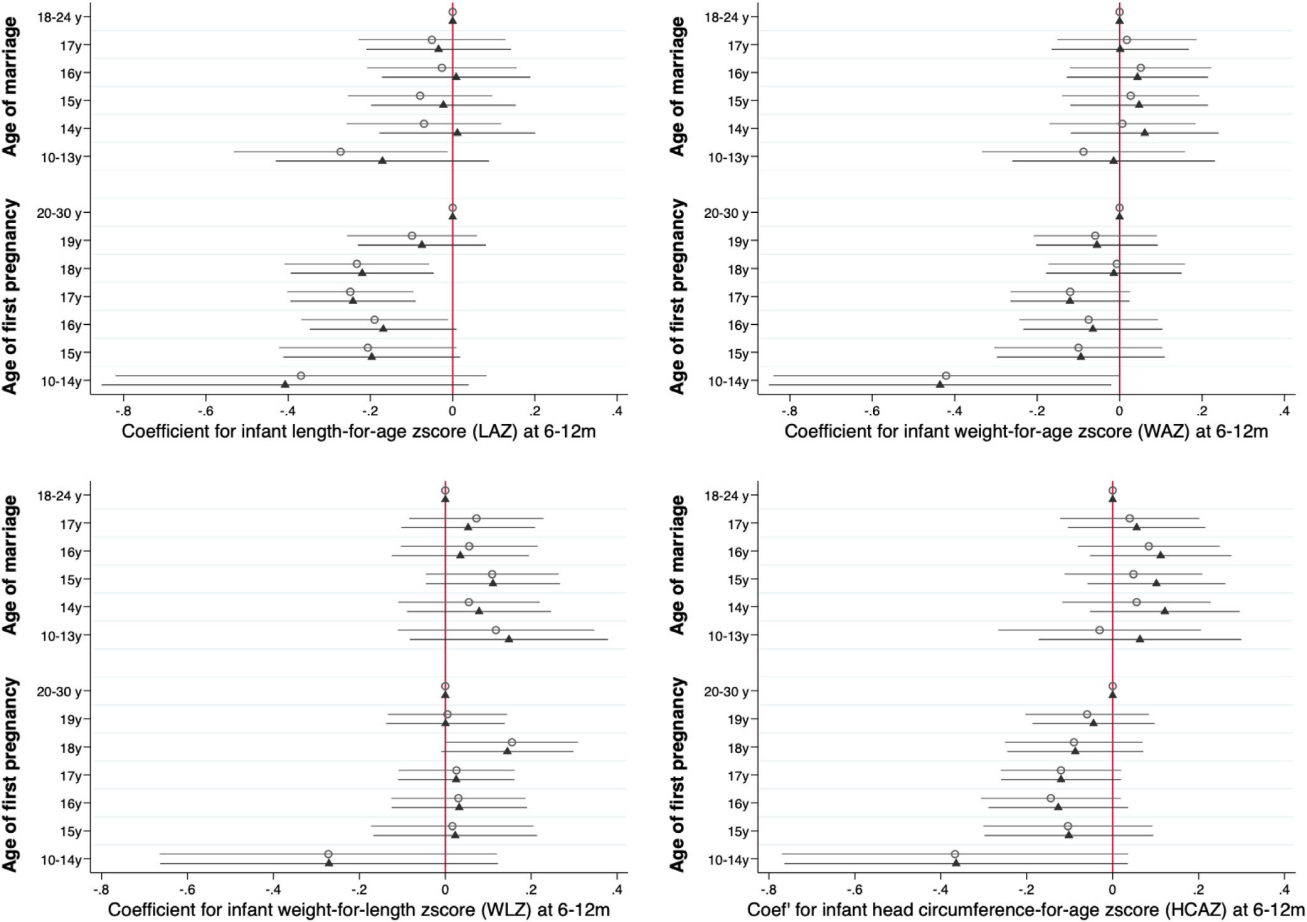
Coefficients from minimally and fully adjusted models of infant anthropometric z‐scores by age at marriage and pregnancy (mutually adjusted) for length‐, weight‐ and head circumference‐for‐age, and weight‐ for‐length z scores between 6 and 12 months. Open circles represent coefficients from models adjusted for clustering using random effects and age in days as a fixed effect. Shaded triangles represent models additionally adjusted for maternal and paternal education, asset tertiles, caste, land ownership, seasonality (cosinor terms) randomization strata, study arm and child sex as fixed effects. Reference categories: Age at marriage 18–24 years; age at first pregnancy 20–30 years (numeric values given in Table S5).

When marriage and pregnancy ages were considered in combination, earlier pregnancy remained associated with lower LAZ and WAZ, with a similar trend to lower HCAZ for those who married at 10–13 years (adjusted ∆ = −0.36, 95% CI –0.76, 0.03) (Figure 5, Table S12, with coefficients for covariates illustrated in Figure S5). Conversely, earlier marriage age broadly showed no associations, except for lower LAZ in those marrying at 10–13 years (adjusted ∆ = −0.17, 95% CI –0.43, 0.09). Adjustment for maternal height slightly attenuated the associations. Regarding covariates, maternal and paternal education, wealth, and membership of advantaged caste were all associated with better infant growth status, while land ownership was also associated with higher HCAZ.

**FIGURE 6:**
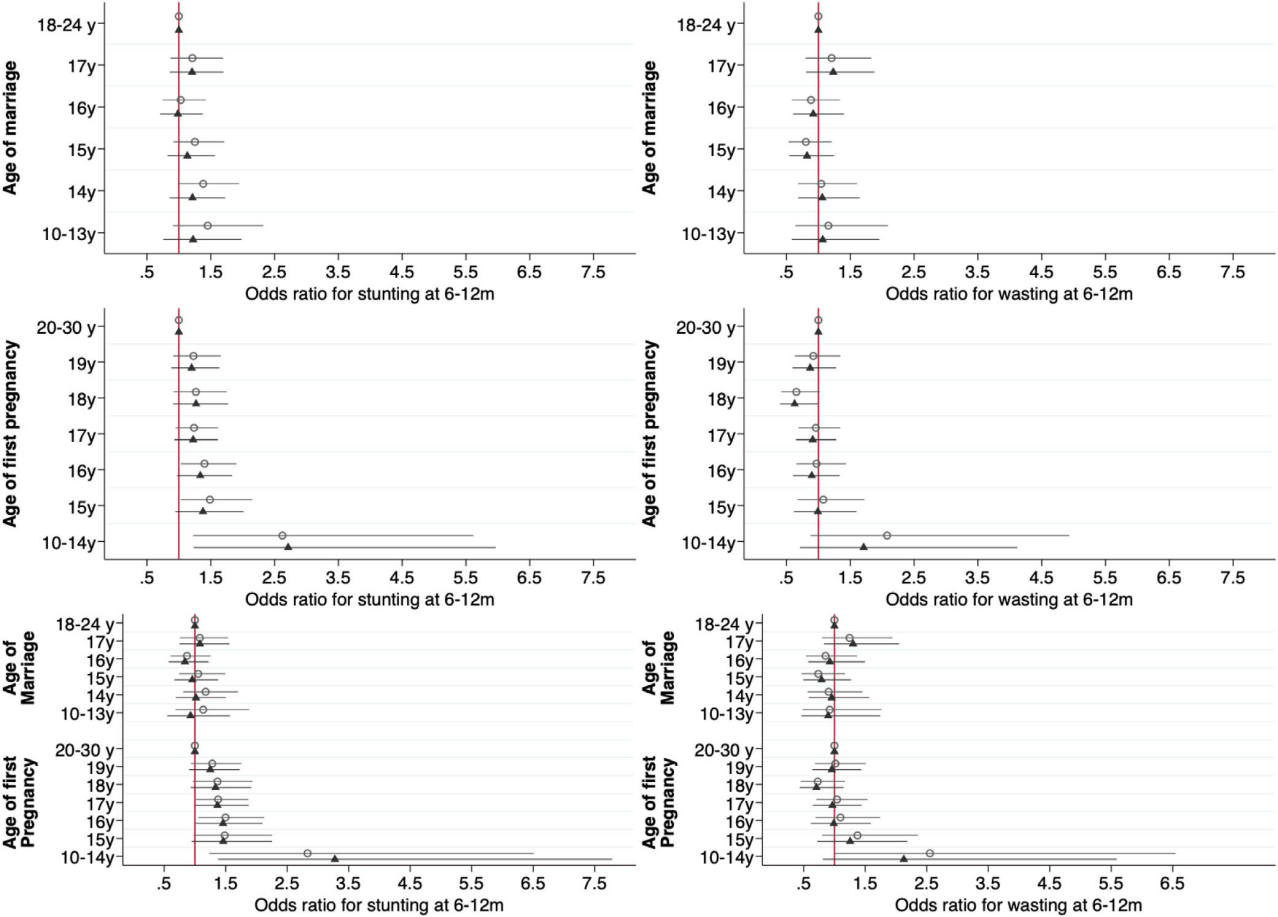
Odds ratios from minimally and fully adjusted logistic regression models of infant stunting and wasting from 180 to 365 days against age at marriage and age at first pregnancy separately and mutually adjusted. Open circles represent odds ratios from models adjusted for clustering using random effects and age in days as a fixed effect only. Shaded triangles represent models additionally adjusted for maternal and paternal education, asset tertiles, caste, land ownership, seasonality (cosinor terms) randomization strata, study arm and child sex as fixed effects. Reference categories: Age at marriage 18–24 years; age at first pregnancy 20–30 years (numeric values given in Table S9).

Figure 6 and Table S13 show equivalent associations for the odds of stunting and wasting, with coefficients for covariates illustrated in Figure S4. Separately, early marriage showed little association with the risk of stunting, whereas pregnancy at 10–13 years was associated with higher risk. Neither marriage age nor pregnancy age was associated with infant wasting. In combined models, early pregnancy at 10–13 years was associated with increased risk of stunting (adjusted OR = 3.28, 95% CI 1.38, 7.78), and this associations changed slightly following additional adjustment for maternal height (adjusted OR = 2.39, 95% CI 0.88, 6.46). After further adjustment for maternal height, pregnancy at 10–13 years remained associated with increased risk of stunting, though the magnitude of effect was attenuated (adjusted OR = 2.39, 95% CI 0.88, 6.46). Although other categories of early pregnancy showed the same pattern in minimally adjusted models, the magnitude of effect was substantially reduced in fully adjusted models. The results for wasting remained null.

### 3.3 Sensitivity analysis

Restricting the sample to women who had marriage before 16 years, we reran the main models investigating associations of early pregnancy with offspring growth. In these analyses, marriage at 14–15 years was the reference category, allowing us to consider whether marriage at 10–13 years had a different association.

For neonatal outcomes, the results were relatively similar to those in the whole sample. Marriage at 10–13 years was not associated with neonatal anthropometry, however earlier pregnancy was associated with lower LAZ, WAZ, WLZ, and HCAZ, with stronger magnitudes of effect, compared to the main sample (Figure S6 and Table S14). Pregnancy at 18, 16, and 15 years was associated with increased odds of stunting (Figure S7 and Table S15), but the associations were not significant, while results for wasting were null.

For infant outcomes, pregnancy at or below 17 years was associated with lower LAZ and HCAZ (Figure S8 and Table S16). In both cases, the magnitude of effect was approximately twice as great for those pregnant at 10–14 years, and these mothers also had infants with lower WAZ and WLZ. These results therefore confirm those illustrated in Figure 4, the main difference being greater deficits in HCAZ associated with early pregnancy in the sensitivity analyses. The risk of stunting was increased among those pregnant at 17 years or earlier, with a large magnitude of effect for those pregnant at 10–13 years (adjusted OR 3.01, 95% CI 1.23, 7.38) (Figure S7 and Table S15). Mothers' pregnancy at 15 years or below also had increased risk of infant wasting, particularly for those pregnant 10–13 years (adjusted OR 2.24, 95% CI 0.79, 6.31). Again, these results confirm those in Figure 5.

## 4 DISCUSSION

Among a population in which the majority of women marry well below the age threshold defined as child marriage by the UN, and who also typically have their first child before 18 years, we found that early marriage and pregnancy among first‐time mothers were both associated with the risk of child undernutrition. Intriguingly, the results showed contrasting patterns for these two exposures, suggesting that marrying early and reproducing early may have different impacts on the capacity of mothers to invest nutritionally in their offspring, potentially due to contrasting underlying pathways. Early pregnancy showed associations with lower LAZ, WAZ and HCAZ that were evident both at birth and in infancy. Independent associations for early marriage were broadly weaker and related primarily to an increased risk of stunting at birth. Our findings indicate that the primary way in which early marriage contributes to infant undernutrition in this population is through acting as a gateway for early reproduction, though early marriage may also impact pregnancy physiology as discussed below.

Our data show that while only a minority of the women who married earliest (10–13 years) reproduced shortly afterwards, those who married at 14 or 15 years had a much greater likelihood of doing so, moreover in the whole sample 55% of the mothers had had their first child by 18 years. Overall, therefore, this is a population where early reproduction remains the norm, in association with other characteristics of the society. We have shown in previous research on this cohort that education is the primary factor associated with delaying marriage to 18+ years (Marphatia et al., 2020), however half of the women in our sample of first‐time mothers had not attended school at all and less than a quarter had attended secondary school.

**FIGURE 7:**
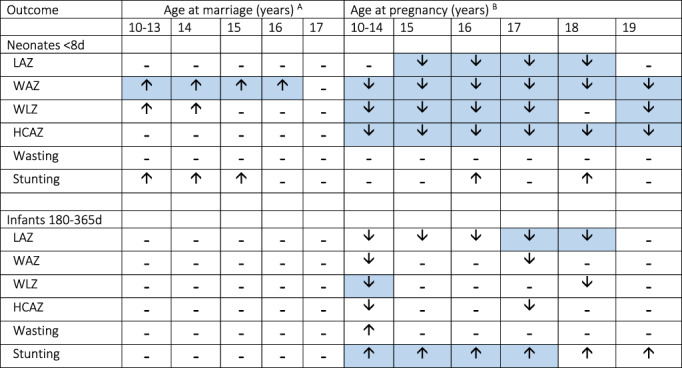
Summary of results for whole analysis using models that include both marriage age and pregnancy age. Stunting defined as LAZ < –2 z‐scores, Wasting defined as WLZ < ‐2 z‐scores. ^A^Coefficients express effect relative to reference group of marriage at 18+ years. ^B^Coefficients express effect relative to reference group of pregnancy at 20+ years. “↓” is lower z‐score value or decreased risk; “↑” is higher z‐score value or increased risk; “**−**” is no association. Blue shaded cells indicate association significant *p <* 0.05, unshaded cells indicate association significant *p <* 0.1. HCAZ, head circumference z‐score; LAZ, length z‐score; WAZ, weight z‐score; WLZ, weight‐for‐length z‐score.

Broadly, we found that early marriage had little effect on neonatal anthropometry, except for a trend towards lower LAZ that translated into an increased odds of stunting. Moreover, a notable trend was that early marriage was associated with increased neonatal WAZ and WLZ, and this trend remained evident for WLZ, though weaker, in infancy. Conversely, early pregnancy was associated more systematically with anthropometric deficits for all neonatal outcomes, with a dose–response association whereby the earlier the pregnancy, the greater the anthropometric deficit. By the second half of infancy, these effects remained evident in markers of growth (LAZ and HCAZ), but less so for markers of nutritional status (WAZ, WLZ) except for the high‐risk group, those pregnant at 10–14 years. Earlier pregnancy was associated with increased risk of neonatal stunting, and this trend became stronger in infancy, particularly those pregnant at 10–14 years. These results are summarized in Figure 7. The magnitudes of effect we describe for both anthropometric z‐scores and the risks of stunting and wasting are of public health importance.

Aiming to go beyond previous research, our statistical models mutually adjusted pregnancy age and marriage age, to identify their independent effects. However, this approach might have given misleading results, as our sample could not include women with high marriage age and low pregnancy age due to the norms for marriage to precede pregnancy in this population. We therefore supported our main models with sensitivity analysis, restricting analysis of pregnancy associations to those who had married before 16 years. These models confirmed the deficits in neonatal and infant anthropometry associated with early pregnancy among those who had married early, which is the common experience in this population.

Our findings show some consistency with previous research, in linking both early reproduction (Fall et al., 2015) and early marriage (Efevbera et al., 2017; Raj et al., 2010; Khan et al., 2019; Prakash et al., 2011) with the risk of poor growth in children, though our approach also differed as we focused on first‐time mothers, and on anthropometric outcomes during both neonatal and infant periods. However, while previous studies on marriage age have tended to control for maternal age at pregnancy, none of them attempted to disentangle the independent associations of marriage and pregnancy age with early growth patterns. Our results indicate that most associations of early marriage (e.g., with WAZ and LAZ) are explained by early reproduction, but also that both early marriage and early pregnancy were independently associated with stunting at birth.

Trying to identify risk factors for early marriage and pregnancy in this population is impractical, because the large majority of girls and women demonstrate both exposures. However, our adjusted analyses indicate that associations of early marriage with neonatal stunting are not due to low household wealth, non‐ownership of land (one marker of food security) or short maternal stature. The fact that the association did not entirely vanish when controlling for early reproduction indicates that potential biological pathways whereby early marriage impacts fetal growth may be different. One possibility is that early marriage is associated with elevated levels of psychosocial stress (John et al., 2019; Khanna et al., 2021), as young brides may be particularly low in the family hierarchy in this setting. However, another possible pathway could be reduced access to food, as wives tend to prepare food and eat last, after other household members have been given their own portions, and often little of the more micronutrient‐rich foods remain for them to eat (Harris‐Fry et al., 2018; Morrison et al., 2018). These pathways may be additive to those that link early reproduction with fetal growth variability, which may relate more to body size and energy and nutrient stores (Scholl et al., 1994). In prior analyses in the same population, both early marriage and early reproduction were independently associated with shorter maternal height (Marphatia et al., 2021b). In exploratory analyses, we found that adjusting for maternal height removed some of the effects, especially for LAZ, which indicates that some of the effects of early marriage and pregnancy upon linear growth in the offspring are explained by variability in maternal size. However, many of the results changed very little after adjustment for maternal height, in particular the association of marriage <16 years with neonatal stunting, and pregnancy at age 10–14 years with infant stunting. This suggests that other biosocial pathways link the exposures with stunting risk.

Intriguingly, when considered together, earlier pregnancy was associated with lower neonatal WAZ, but earlier marriage with higher WAZ values. It is possible that women who marry at very young age differ in their nutritional status, which might then influence the decision of the natal and marital households to agree the marriage. Relevant to this, an earlier analysis of maternal BMI in 35 African countries found that after adjusting for early childbearing, early marriage itself was associated with a reduced risk of the mother having low BMI (Efevbera et al., 2019), though the findings from an Indian study were in the opposite direction (Prakash et al., 2011). Research in India has linked earlier menarche with earlier marriage (Raj et al., 2015), and earlier menarche may be associated with higher BMI (Malitha et al., 2020; Żegleń et al., 2020). If women marrying relatively young in our population do indeed have higher BMI, this might also help explain why for some outcomes, those who gave birth at the earliest age (10–14 years) did not always have the worst offspring anthropometric outcomes.

The fact that associations were stronger in the neonatal compared to the later infant period suggests that the physiology of pregnancy may be more sensitive to the stresses of early marriage and pregnancy compared to lactation. To some extent, the weaker effects in infancy may indicate the potential for catch‐up growth after birth, potentially because lactation can more readily be funded by body fat stores than placental nutrition (Ellison, 2008), which is most closely associated with maternal metabolic rate (Aldoretta & Hay Jr., 1995; Wells, 2018). Thus, fetal growth may track maternal dietary intake and its psychosocial influences more closely than does infant growth, and both early marriage and early pregnancy may impact the mother's diet more than her somatic energy stores. Of relevance here, our findings indicate that the main effects of early pregnancy on infants are deficits in growth markers (LAZ, HCAZ), suggesting that infants have recovered from earlier deficits in nutritional status (WAZ, WLZ) by allocating energy from linear growth. We did not test this recovery prospectively in our study, but such a pattern has been reported previously in a longitudinal study of Gambian infants (Schoenbuchner et al., 2019).

Overall, our analysis provides evidence for the composite role of maternal capital in supporting growth of the offspring in early life (Wells, 2010). Both early marriage, as a proxy for mother's position in the family hierarchy (social capital), and early pregnancy, as a proxy for aspects of reproductive physiology (somatic capital), are associated with the risk of child undernutrition. Notably, models showed relatively small changes in coefficients between minimally and fully adjusted models. This suggests that the patterns we have identified are not due to residual confounding, and that the timing of women's marriage and pregnancy directly impacts the growth of the next generation. Our findings therefore support the UNICEF (1990) model, in showing that women's experience of both marriage and pregnancy contribute to the link between societal norms and practices and child undernutrition, but with pregnancy playing the primary role in this population.

The strengths of the study include the availability of data on a relatively large sample of women from a disadvantaged setting, enabling us to look beyond early marriage and reproduction per se, to examine whether the impacts on children worsen, the earlier these events occur in a woman's adolescence. We also benefitted from data on a range of relevant confounders, measured prospectively before the birth of the child. Having demonstrated that maternal and infant anthropometry demonstrate seasonality in this population (Saville et al., 2022a), we adjusted for this source of environmental stress. Although our sample of primigravid women was broadly representative of first‐time mothers with young children in Province 2 of Nepal, these mothers are somewhat better‐off and better‐educated than multigravid mothers. Finally, as we did not study multigravid women, we do not know whether early marriage is associated with effects on the growth patterns of subsequent children. However, concentrating upon primigravidae enabled us to examine the effects of early marriage and pregnancy without the “noise” generated by factors associated with previous child‐bearing.

Limitations include the fact that we analyzed data from a cluster‐randomized trial, in which some study arms were associated with differences in birth anthropometry (Saville et al., 2018). However, we adjusted for trial arm in all models, and consider that our findings are not confounded by the intervention. There was a large amount of missing data on maternal anthropometry, preventing us from examining in detail the extent to which these associations are explained by variability in maternal BMI. We lacked life‐course data on relevant factors such as maternal age at menarche. We also did not examine the multiparous women in this sample, because the majority of their children in the trial (with anthropometric data) were higher parity children and we suspect that the relationships between ages at marriage and pregnancy on the one hand and infant outcomes on the other may well be different for higher parity infants, confounded by complex factors such as maternal age, family size and inter‐birth interval. Finally, though we treated the data as cross‐sectional, almost a quarter of the sample (22%) were measured at both time points, reducing the independence of the analyses of neonatal and infant size due to the influence of growth trajectory. Only 7 infants were measured twice between 180 and 365 days.

## 5 CONCLUSION

In a population from lowland Nepal where a large majority of women marry and reproduce much earlier than is considered lawful by the Nepal government (Government of Nepal, 2017; His Majesty's Government of Nepal, 1963), both early marriage and early pregnancy are associated with worse anthropometric outcomes of the firstborn offspring. The associations are driven primarily by early reproduction, indicating that early marriage functions primarily as a gateway for early reproduction, though for LAZ at birth the two exposures demonstrate independent associations. The associations are also more evident among neonates than in older infants, suggesting that pregnancy is the period in which maternal nutrition is more susceptible to these stresses. However, in infancy, deficits in growth markers (LAZ and HCAZ) associated with early pregnancy remain apparent. Further work may elucidate in more detail the possible biological and social mechanisms that may underlie these associations.

## AUTHOR CONTRIBUTIONS

Jonathan C. K. Wells and Naomi M. Saville conceived the paper and the analyses. Naomi M. Saville led data collection on the ground, conducted all the analyses, and prepared all figures and tables. Jonathan C. K. Wells wrote the first draft of the paper for publication. Dharma S. Manandhar managed the field data collection team in Nepal. All authors reviewed the manuscript, provided input, and approved the paper for publication.

## CONFLICT OF INTEREST

The authors declare no conflicts of interest.

## Supporting information


**Appendix S1**: Supporting Information.

## Data Availability

The data that support the findings of this study are available upon reasonable request from Naomi Saville (n.saville@ucl.ac.uk).
