# Supplementary material for: Associations of maternal age at marriage and pregnancy with infant undernutrition: Evidence from first‐time mothers in rural lowland Nepal
Source: Am J Biol Anthropol. 2022 May 24;178(4):557–73. doi: 10.1002/ajpa.24560 (PMC9539981; doi:10.1002/ajpa.24560)
Supplement: Supplementary file 1 — Appendix S1: Supporting Information. [file AJPA-178-557-s001.docx]

**Wells JC, Marphatia AA, Cortina-Borja M, Manandhar DS, Reid AM, Saville NM. Associations of maternal age at marriage and pregnancy with infant undernutrition: evidence from first-time mothers in rural lowland Nepal**

**Supplementary material**

**Figure S1. Study flow chart**

**Figure S2. Coefficients from minimally and fully adjusted full models of length-for-age z-score in the first 8 days of life by age at marriage and pregnancy mutually adjusted, showing all covariates.**


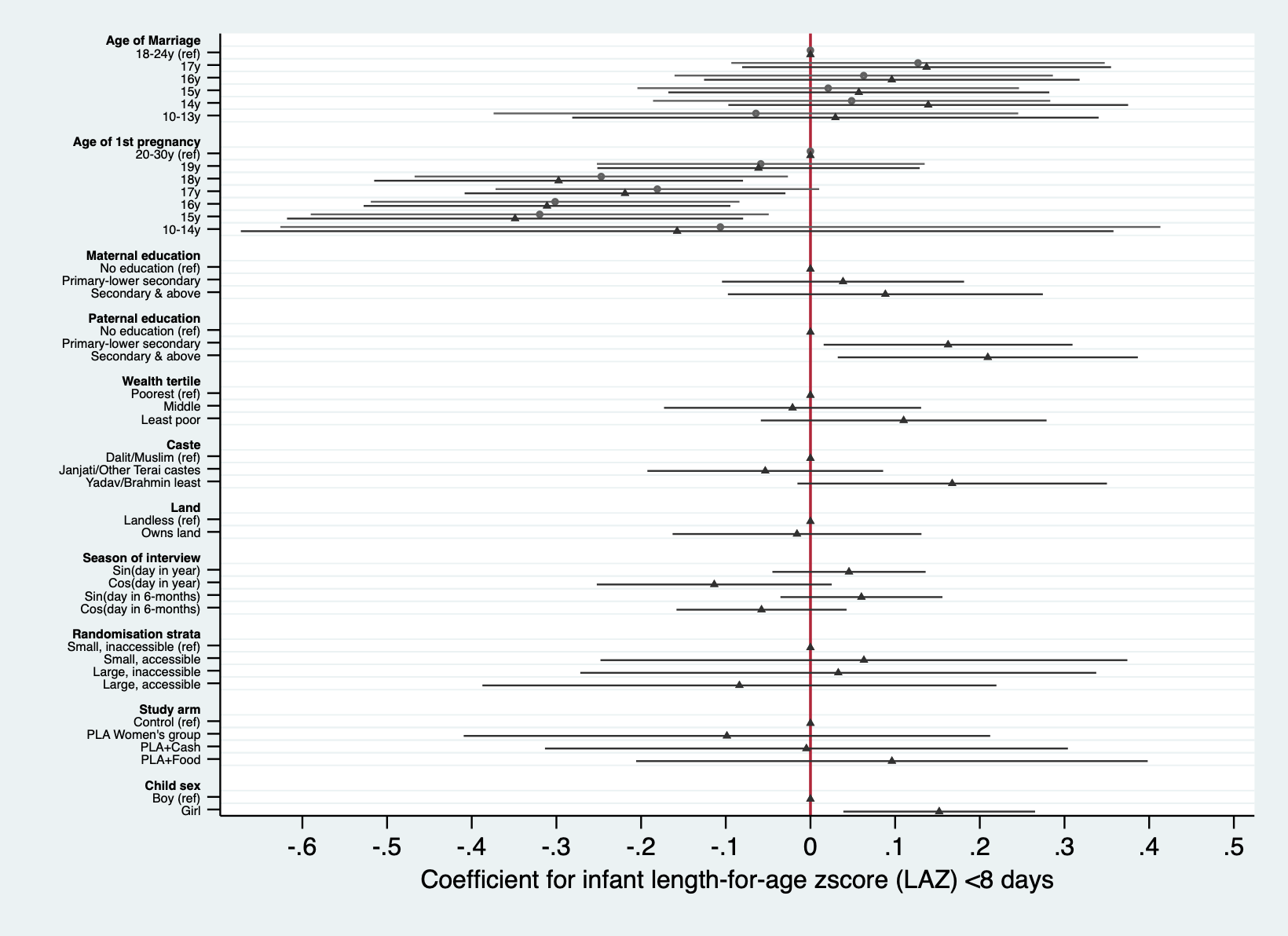


Legend: Circles represent coefficients from models adjusted for clustering using random effects only. Triangles represent coefficients from models additionally adjusted for maternal and paternal education, asset tertiles, caste, land ownership, seasonality (cosinor terms), randomization strata, study arm and child sex as fixed effects. Reference categories for each variable are indicated on the y-axis.

All analyses involved similar adjustments for covariates.

**Figure S3. Coefficients from minimally and fully adjusted full models of anthropometric z-scores in the first 8 days of life by age at marriage and pregnancy mutually adjusted, showing socio-economic covariates**


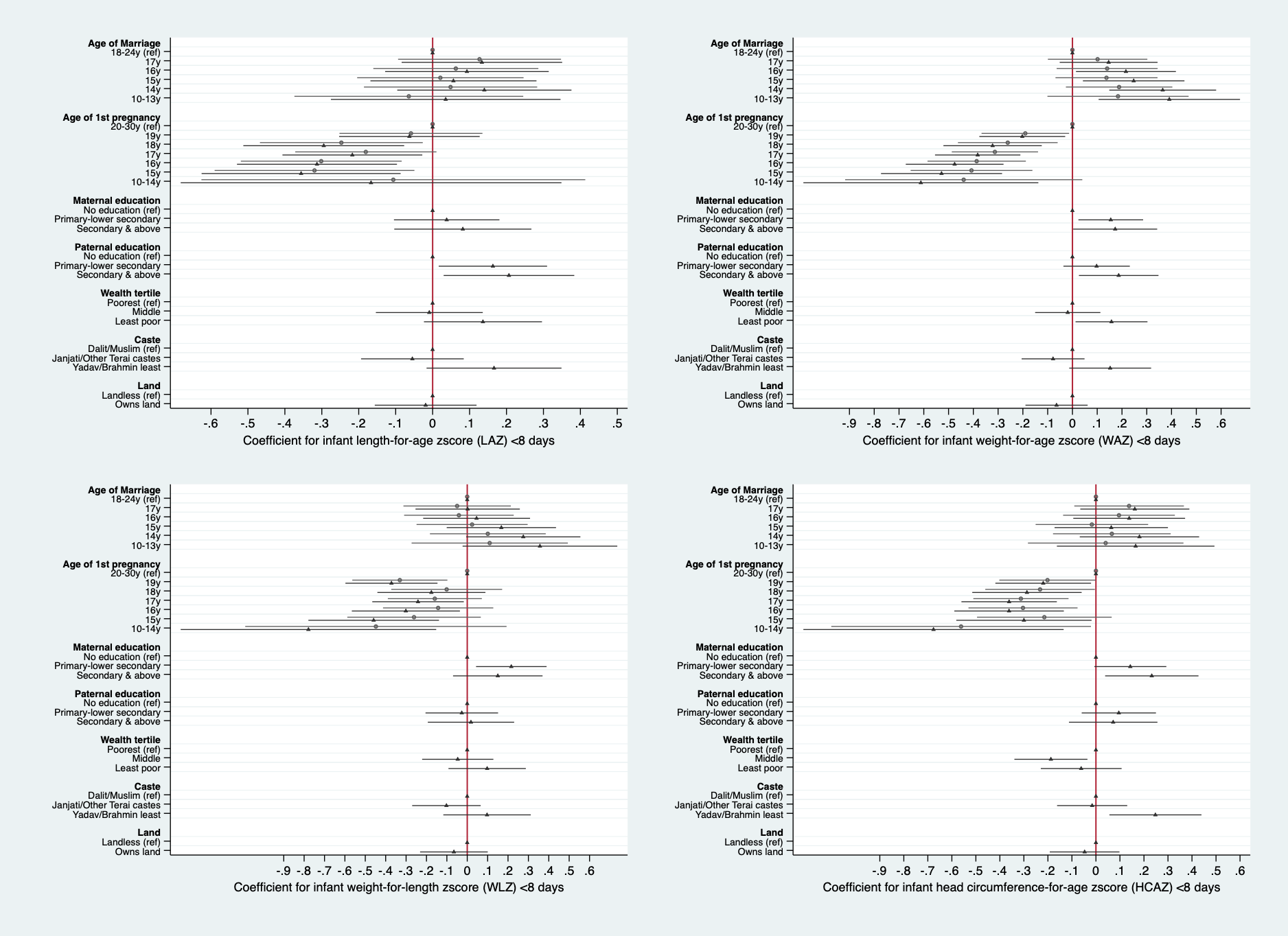


**Figure S4. Coefficients from minimally and fully adjusted full models of wasting and stunting in the first 8 days and second six months of life by age at marriage and pregnancy mutually adjusted, showing socio-economic covariates**

**
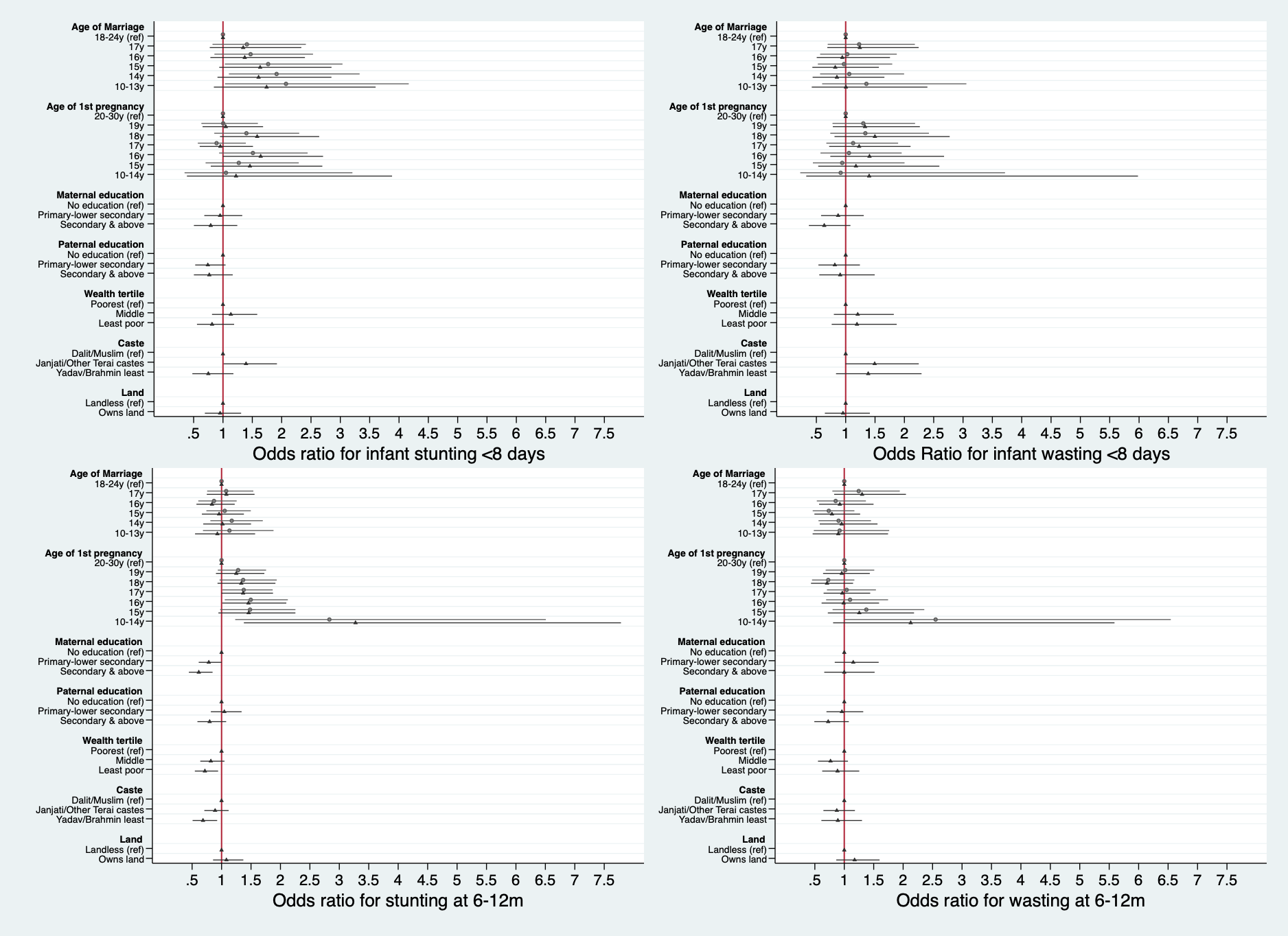
**

**Figure S5. Coefficients from minimally and fully adjusted full models of anthropometric z-scores in the second 6 months of life by age at marriage and pregnancy mutually adjusted, showing socio-economic covariates**

**
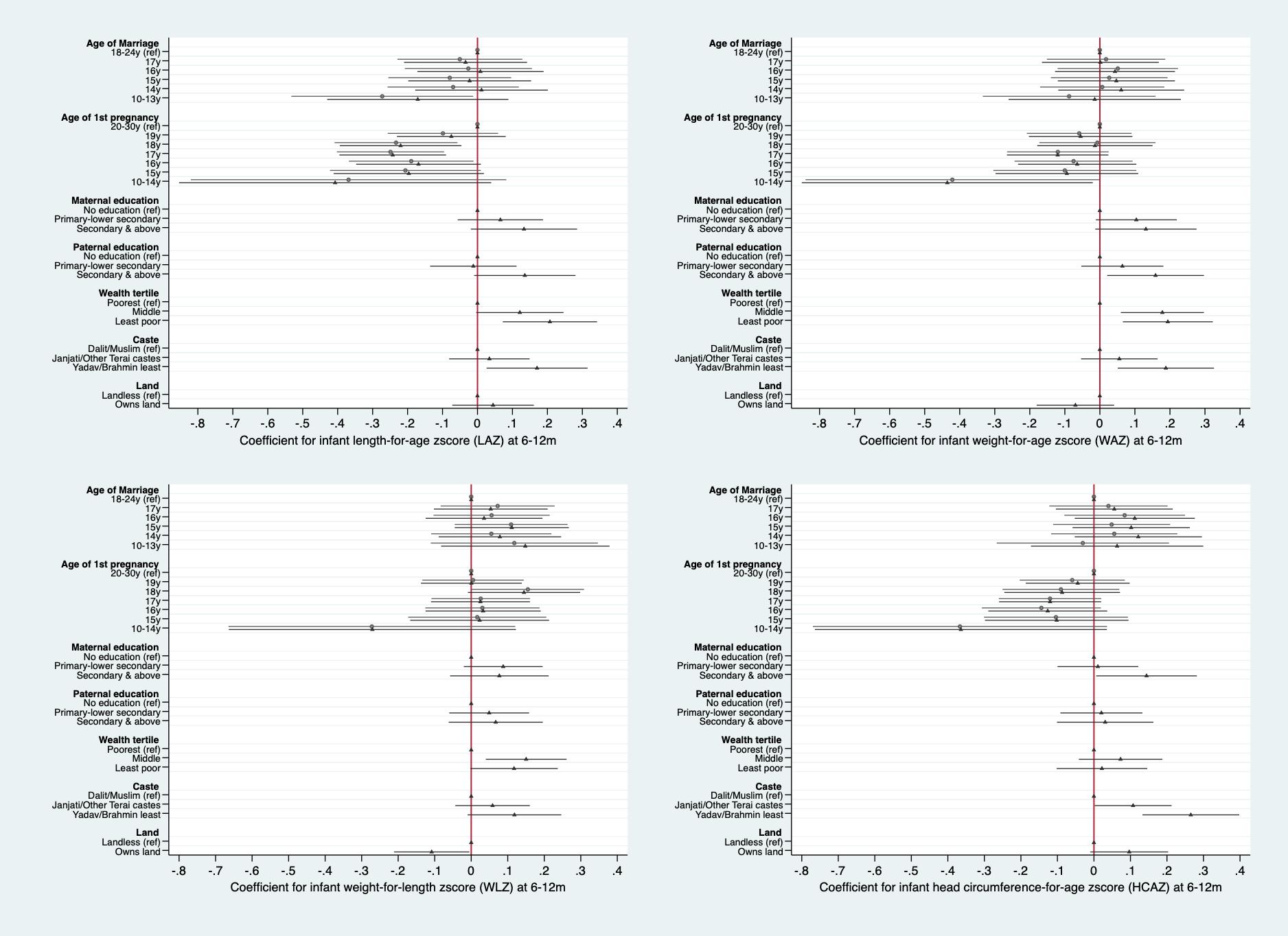
**

**Figure S6. Coefficients from minimally and fully adjusted linear regression models anthropometric z scores (HAZ, WAZ, WLZ and HCZ) in the first 8 days of life against age at marriage and age at first pregnancy mutually adjusted amongst neonates of mothers who married below 16 years.** Numeric values given in Table S10.

**
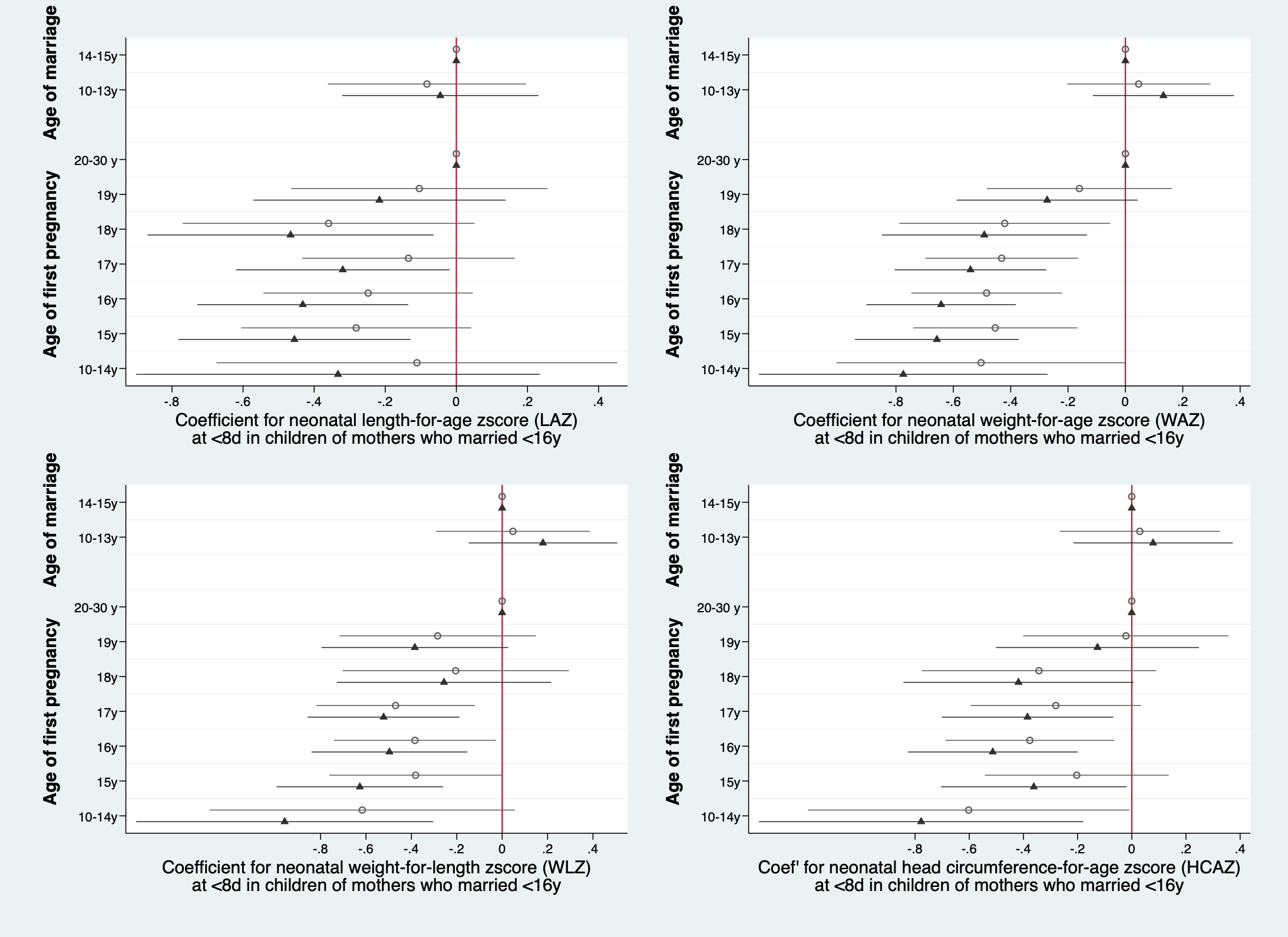
**

Legend: Open circles represent odds ratios from models adjusted for clustering using random effects and age in days as a fixed effect only. Shaded triangles represent models additionally adjusted for maternal and paternal education, asset tertiles, caste, land ownership, seasonality (cosinor terms) randomization strata, study arm and child sex as fixed effects. Reference categories: age at marriage 14-15 years; age at first pregnancy 20-30 years.

**Figure S7. Odds ratios from minimally and fully adjusted logistic regression models of stunting and wasting in the first 8 days of life and from 180 to 365 days against age at marriage and age at first pregnancy mutually adjusted amongst neonates and infants of mothers who married below 16 years.** Numeric values given in Table S12.


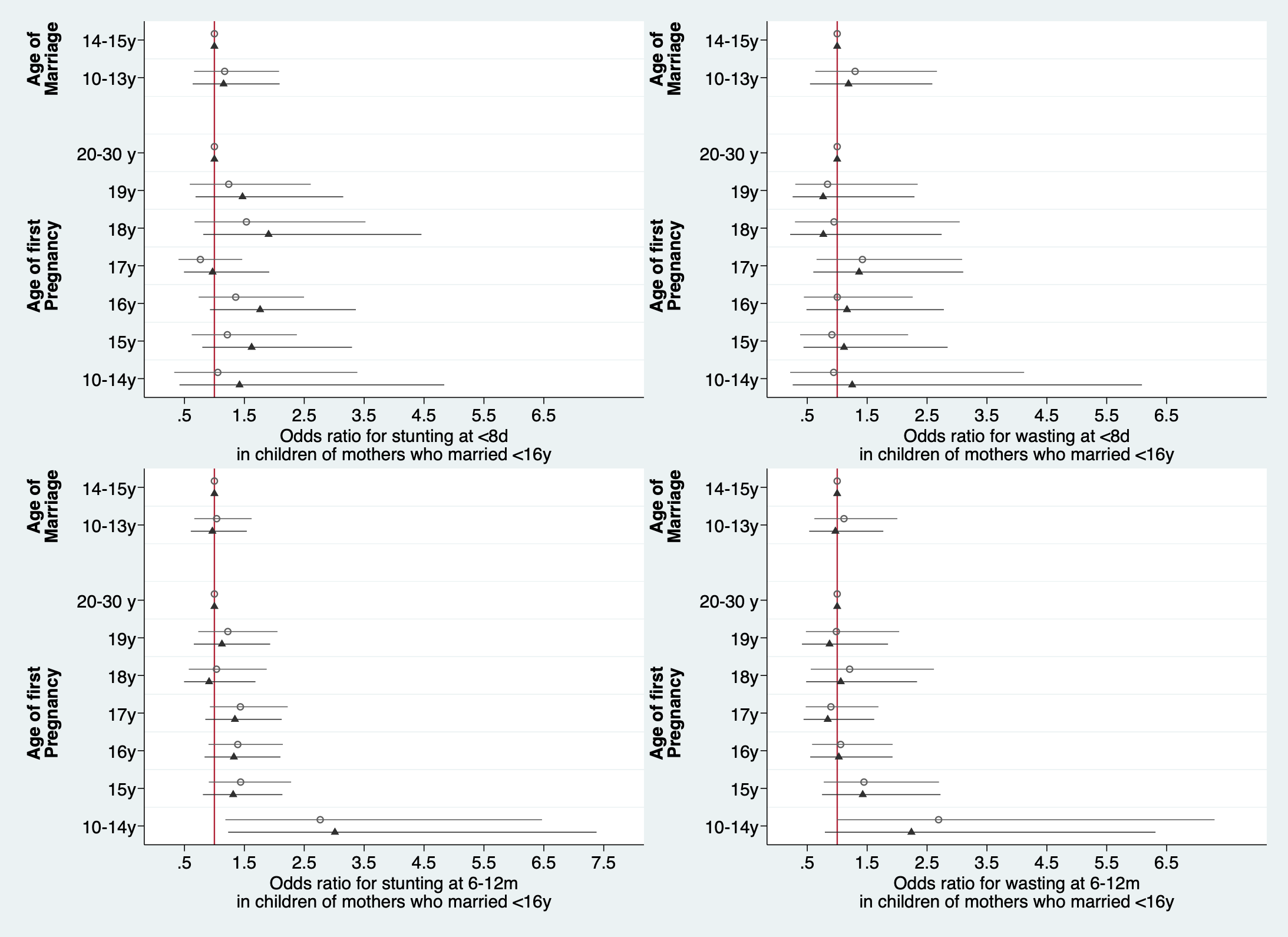


Legend: Open circles represent odds ratios from models adjusted for clustering using random effects and age in days as a fixed effect only. Shaded triangles represent models additionally adjusted for maternal and paternal education, asset tertiles, caste, land ownership, seasonality (cosinor terms) randomization strata, study arm and child sex as fixed effects. Reference categories: age at marriage 14-15 years; age at first pregnancy 20-30 years.

**Figure S8. Coefficients from minimally and fully adjusted linear regression models of anthropometric z scores (HAZ, WAZ, WLZ and HCZ) from 180 to 365 days against age at marriage and age at first pregnancy mutually adjusted amongst infants of mothers who married below 16 years.** Numeric values given in Table S11.

**
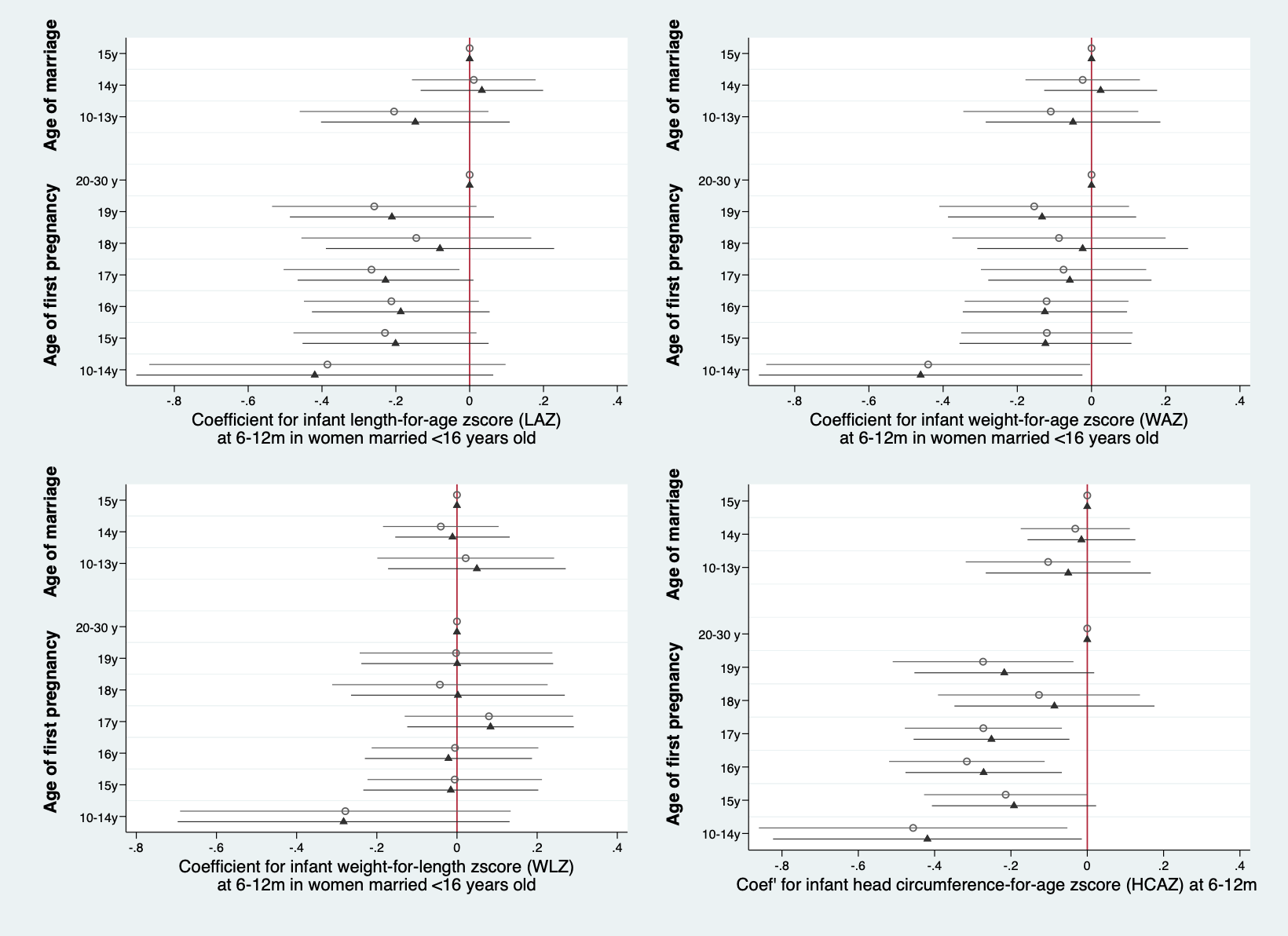
**

Legend: Open circles represent odds ratios from models adjusted for clustering using random effects and age in days as a fixed effect only. Shaded triangles represent models additionally adjusted for maternal and paternal education, asset tertiles, caste, land ownership, seasonality (cosinor terms) randomization strata, study arm and child sex as fixed effects. Reference categories: age at marriage 14-15 years; age at first pregnancy 20-30 years.

**Table S1. Socioeconomic variables by caste**

**
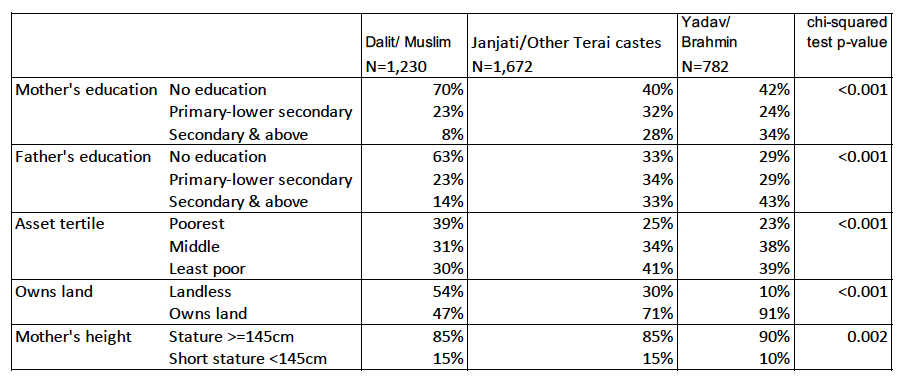
**

**Table S2. Characteristics of primigravida women compared with multiparous women and, sampled primigravida women compared with not sampled primigravidae.**

| **Characteristics** | **Multigravidae** | | **Primigravidae** | | chi-squared *p* value | **Primigravidae not sampled** | | **Primigravidae sampled** | | chi-squared *p* value |
| --- | --- | --- | --- | --- | --- | --- | --- | --- | --- | --- |
|  | N=15,195 | | N=8,544 | |  | N=5,542 | | N=3,002 | |  |
|  | Freq. | % | Freq. | % |  | Freq. | % | Freq. | % |  |
| **Age at marriage (18-24 ref)** | 13,182 |  | 7,603 |  | <0.001 | 4,821 |  | 2,782 |  | 0.37 |
| 10-13y | 2,045 | 15.5% | 365 | 4.8% |  | 219 | 4.5% | 146 | 5.2% |  |
| 14y | 3,359 | 25.5% | 1,134 | 14.9% |  | 696 | 14.4% | 438 | 15.7% |  |
| 15y | 3,544 | 26.9% | 1,847 | 24.3% |  | 1,169 | 24.2% | 678 | 24.4% |  |
| 16y | 1,714 | 13.0% | 1,738 | 22.9% |  | 1,120 | 23.2% | 618 | 22.2% |  |
| 17y | 1,381 | 10.5% | 1,381 | 18.2% |  | 883 | 18.3% | 498 | 17.9% |  |
| 18-24 y | 1,139 | 8.6% | 1,138 | 15.0% |  | 734 | 15.2% | 404 | 14.5% |  |
| **Age 1st pregnancy (20-30 ref)** | 14,673 |  | 8,544 |  | <0.001 | 5,542 |  | 3,002 |  | 0.15 |
| 10-14y | 1,383 | 9.4% | 119 | 1.4% |  | 77 | 1.4% | 42 | 1.4% |  |
| 15y | 2,317 | 15.8% | 626 | 7.3% |  | 394 | 7.1% | 232 | 7.7% |  |
| 16y | 2,761 | 18.8% | 1,214 | 14.2% |  | 770 | 13.9% | 444 | 14.8% |  |
| 17y | 2,752 | 18.8% | 2,251 | 26.3% |  | 1,443 | 26.0% | 808 | 26.9% |  |
| 18y | 1,988 | 13.5% | 1,165 | 13.6% |  | 796 | 14.4% | 369 | 12.3% |  |
| 19y | 1,614 | 11.0% | 1,379 | 16.1% |  | 888 | 16.0% | 491 | 16.4% |  |
| 20-30 y | 1,858 | 12.7% | 1,790 | 21.0% |  | 1,174 | 21.2% | 616 | 20.5% |  |
| **Mother education** | 15,195 |  | 8,521 |  | <0.001 | 5,521 |  | 3,000 |  | 0.060 |
| never went to school | 11,020 | 72.5% | 4,177 | 49.0% |  | 2,688 | 48.7% | 1,489 | 49.6% |  |
| primary to lower secondary | 2,545 | 16.7% | 2,280 | 26.8% |  | 1,452 | 26.3% | 828 | 27.6% |  |
| secondary and above | 1,630 | 10.7% | 2,064 | 24.2% |  | 1,381 | 25.0% | 683 | 22.8% |  |
| **Husband education** | 15,194 |  | 8,521 |  | <0.001 | 5,521 |  | 3,000 |  | 0.079 |
| never went to school | 7,948 | 52.3% | 3,477 | 40.8% |  | 2,216 | 40.1% | 1,261 | 42.0% |  |
| primary to lower secondary | 3,996 | 26.3% | 2,419 | 28.4% |  | 1,560 | 28.3% | 859 | 28.6% |  |
| secondary and above | 3,250 | 21.4% | 2,625 | 30.8% |  | 1,745 | 31.6% | 880 | 29.3% |  |
| **Asset tertile (11 assets)** | 15,021 |  | 8,436 |  | <0.001 | 5,454 |  | 2,982 |  | 0.88 |
| Poorest | 5,513 | 36.7% | 2,400 | 28.4% |  | 1,542 | 28.3% | 858 | 28.8% |  |
| Middle | 4,936 | 32.9% | 2,876 | 34.1% |  | 1,862 | 34.1% | 1,014 | 34.0% |  |
| Least poor | 4,572 | 30.4% | 3,160 | 37.5% |  | 2,050 | 37.6% | 1,110 | 37.2% |  |
| **Caste** | 15,195 |  | 8,544 |  | <0.001 | 5,542 |  | 3,002 |  | 0.010 |
| Dalit/Muslim disadvantaged | 5,643 | 37.1% | 2,837 | 33.2% |  | 1,835 | 33.1% | 1,002 | 33.4% |  |
| Janjati/Other Terai castes middle | 6,303 | 41.5% | 3,677 | 43.0% |  | 2,336 | 42.2% | 1,341 | 44.7% |  |
| Yadav/Brahmin least disadvantaged | 3,249 | 21.4% | 2,030 | 23.8% |  | 1,371 | 24.7% | 659 | 22.0% |  |
| **Owns land** | 15,195 |  | 8,518 |  | <0.001 | 5,520 |  | 2,998 |  | 0.68 |
| No | 5,406 | 35.6% | 2,760 | 32.4% |  | 1,780 | 32.2% | 980 | 32.7% |  |
| Yes | 9,789 | 64.4% | 5,758 | 67.6% |  | 3,740 | 67.8% | 2,018 | 67.3% |  |
| **Randomisation stratum** | 15,195 |  | 8,544 |  | <0.001 | 5,542 |  | 3,002 |  | 0.002 |
| small, inaccessible | 3,142 | 20.7% | 1,768 | 20.7% |  | 1,085 | 19.6% | 683 | 22.8% |  |
| small, accessible | 3,314 | 21.8% | 1,922 | 22.5% |  | 1,240 | 22.4% | 682 | 22.7% |  |
| large, inaccessible | 4,522 | 29.8% | 2,341 | 27.4% |  | 1,565 | 28.2% | 776 | 25.8% |  |
| large, accessible | 4,217 | 27.8% | 2,513 | 29.4% |  | 1,652 | 29.8% | 861 | 28.7% |  |
| **Study arm woman enrolled in** | 15,195 |  | 8,544 |  | 0.72 | 5,542 |  | 3,002 |  | <0.001 |
| Control | 3,226 | 21.2% | 1,867 | 21.9% |  | 1,172 | 21.1% | 695 | 23.2% |  |
| Women's group | 3,440 | 22.6% | 1,931 | 22.6% |  | 1,268 | 22.9% | 663 | 22.1% |  |
| Cash | 4,397 | 28.9% | 2,452 | 28.7% |  | 1,664 | 30.0% | 788 | 26.2% |  |
| Food | 4,132 | 27.2% | 2,294 | 26.8% |  | 1,438 | 25.9% | 856 | 28.5% |  |
| **Sex of infant** | 12,566 |  | 6,637 |  | 0.023 | 3,635 |  | 3,002 |  | 0.46 |
| Boy | 6,713 | 53.4% | 3,431 | 51.7% |  | 1,864 | 51.3% | 1,567 | 52.2% |  |
| Girl | 5,853 | 46.6% | 3,206 | 48.3% |  | 1,771 | 48.7% | 1,435 | 47.8% |  |
| **Mother's height** | 10,856 |  | 5,103 |  | <0.001 | 2,793 |  | 2,310 |  | 0.87 |
| <145cm | 1,761 | 16.2% | 719 | 14.1% |  | 400 | 14.3% | 319 | 13.8% |  |
| 145 - 147.9cm | 1,783 | 16.4% | 800 | 15.7% |  | 438 | 15.7% | 362 | 15.7% |  |
| >=148cm | 7,312 | 67.4% | 3,584 | 70.2% |  | 1,955 | 70.0% | 1,629 | 70.5% |  |

**Table S3. Age at marriage and socioeconomic or maternal variables**

**
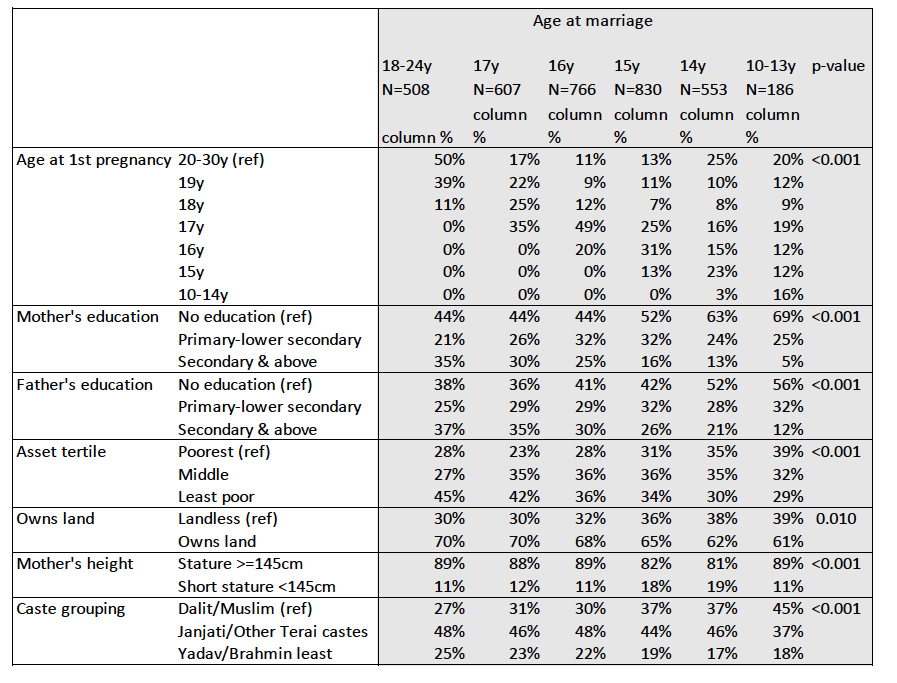
**

**Table S4. Age at first pregnancy and socioeconomic or maternal variables**

**
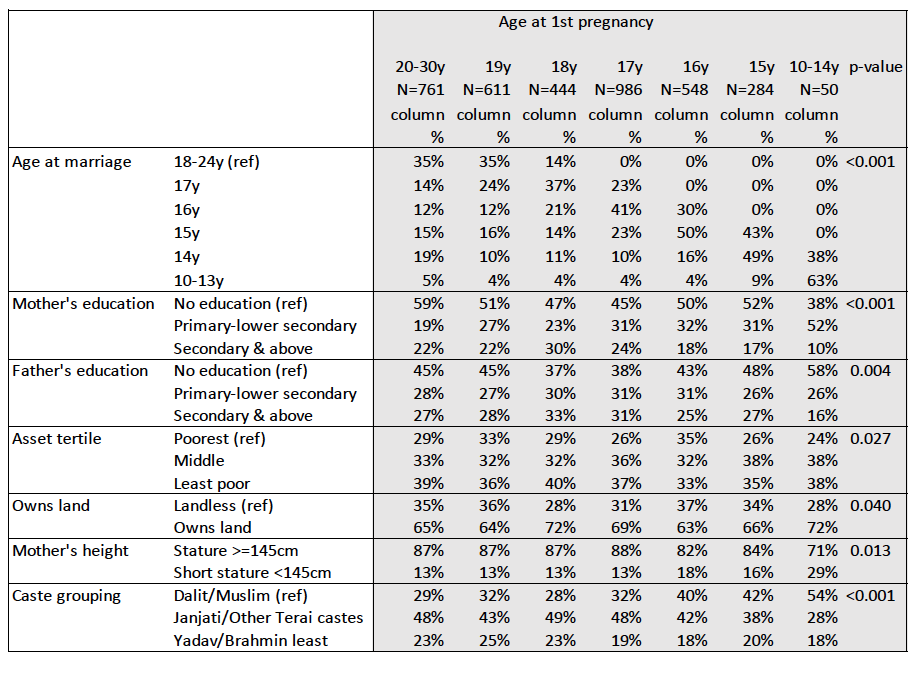
**

**Table S5. Age at marriage and pregnancy by caste**

**
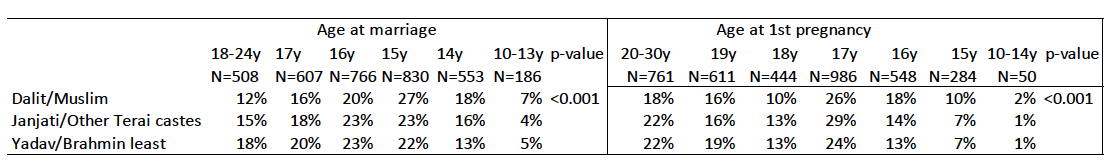
**

**Table S6.** **Coefficients from minimally and fully adjusted models of neonatal anthropometric z-scores by age at marriage for length-, weight- and head circumference-for-age, and weight- for-length z scores in the first 8 days of life, showing models minimally adjusted, and adjusted with and without height of mother in the model.**

|  |  | **Model 1: Minimally adjusted (only cluster)** | | | | **Model 2: Adjusted (multiple covariates without mother's height)** | | | | **Model 3: Adjusted (multiple covariates including mother's height)** | | | |
| --- | --- | --- | --- | --- | --- | --- | --- | --- | --- | --- | --- | --- | --- |
| Outcome | Age married | n | Coeff. | (95% CI) | *p* value | n | Coeff. | (95% CI) | *p* value | n | Coeff. | (95% CI) | p value |
| Length-for-age z score <8 days | 10-13 y |  | -0.170 | (-0.458, 0.119) | 0.2494 |  | -0.099 | (-0.388, 0.19) | 0.5009 |  | -0.101 | (-0.429, 0.227) | 0.5455 |
|  | 14 y |  | -0.083 | (-0.299, 0.133) | 0.4530 |  | -0.015 | (-0.231, 0.202) | 0.8940 |  | 0.105 | (-0.139, 0.349) | 0.3996 |
|  | 15 y |  | -0.140 | (-0.338, 0.059) | 0.1672 |  | -0.124 | (-0.321, 0.074) | 0.2198 |  | -0.063 | (-0.292, 0.165) | 0.5868 |
|  | 16 y |  | -0.067 | (-0.265, 0.131) | 0.5079 |  | -0.058 | (-0.255, 0.138) | 0.5610 |  | -0.043 | (-0.266, 0.181) | 0.7091 |
|  | 17 y |  | 0.038 | (-0.171, 0.247) | 0.7215 |  | 0.024 | (-0.182, 0.23) | 0.8203 |  | 0.033 | (-0.2, 0.266) | 0.7812 |
|  | 18-24 y (ref) | 1346 | 0.000 |  |  | 1345 | 0 |  |  | 961 | 0 |  |  |
| Weight-for-age z score <8 days | 10-13 y |  | 0.019 | (-0.247, 0.285) | 0.8875 |  | 0.152 | (-0.114, 0.419) | 0.2620 |  | 0.260 | (-0.042, 0.562) | 0.0914 |
|  | 14 y |  | 0.035 | (-0.163, 0.233) | 0.7304 |  | 0.147 | (-0.051, 0.345) | 0.1459 |  | 0.189 | (-0.035, 0.413) | 0.0983 |
|  | 15 y |  | -0.056 | (-0.237, 0.126) | 0.5477 |  | -0.006 | (-0.187, 0.175) | 0.9487 |  | 0.049 | (-0.16, 0.258) | 0.6472 |
|  | 16 y |  | -0.022 | (-0.204, 0.16) | 0.8116 |  | 0.006 | (-0.174, 0.186) | 0.9460 |  | 0.049 | (-0.156, 0.253) | 0.6415 |
|  | 17 y |  | -0.006 | (-0.197, 0.185) | 0.9489 |  | 0.007 | (-0.182, 0.195) | 0.9428 |  | 0.043 | (-0.171, 0.257) | 0.6939 |
|  | 18-24 y (ref) | 1369 | 0.000 |  |  | 1368 | 0 |  |  | 980 | 0 |  |  |
| Weight-for-length z score <8 days | 10-13 y |  | 0.047 | (-0.309, 0.403) | 0.7963 |  | 0.193 | (-0.158, 0.545) | 0.2809 |  | 0.325 | (-0.087, 0.738) | 0.1225 |
|  | 14 y |  | 0.088 | (-0.171, 0.348) | 0.5047 |  | 0.180 | (-0.075, 0.435) | 0.1674 |  | 0.138 | (-0.157, 0.433) | 0.3593 |
|  | 15 y |  | 0.005 | (-0.235, 0.245) | 0.9680 |  | 0.072 | (-0.164, 0.307) | 0.5518 |  | 0.047 | (-0.235, 0.329) | 0.7457 |
|  | 16 y |  | -0.041 | (-0.279, 0.197) | 0.7355 |  | -0.009 | (-0.24, 0.223) | 0.9415 |  | -0.052 | (-0.324, 0.221) | 0.7093 |
|  | 17 y |  | -0.069 | (-0.318, 0.181) | 0.5886 |  | -0.047 | (-0.29, 0.196) | 0.7057 |  | -0.062 | (-0.345, 0.222) | 0.6703 |
|  | 18-24 y (ref) | 1175 | 0.000 |  |  | 1174 | 0 |  |  | 838 | 0 |  |  |
| Head circumference-for-age z score <8 days | 10-13 y |  | -0.108 | (-0.41, 0.194) | 0.4825 |  | -0.032 | (-0.337, 0.272) | 0.8350 |  | 0.070 | (-0.284, 0.423) | 0.6993 |
|  | 14 y |  | -0.032 | (-0.257, 0.194) | 0.7839 |  | 0.043 | (-0.185, 0.271) | 0.7125 |  | 0.120 | (-0.143, 0.382) | 0.3709 |
|  | 15 y |  | -0.153 | (-0.36, 0.053) | 0.1461 |  | -0.110 | (-0.317, 0.098) | 0.3002 |  | 0.018 | (-0.226, 0.263) | 0.8829 |
|  | 16 y |  | -0.048 | (-0.255, 0.159) | 0.6483 |  | -0.036 | (-0.242, 0.171) | 0.7352 |  | 0.019 | (-0.22, 0.259) | 0.8738 |
|  | 17 y |  | 0.038 | (-0.179, 0.255) | 0.7293 |  | 0.041 | (-0.175, 0.257) | 0.7089 |  | 0.148 | (-0.102, 0.398) | 0.2455 |
|  | 18-24 y (ref) | 1347 | 0.000 |  |  | 1346 | 0 |  |  | 962 | 0 |  |  |

Legend: Model 1 minimally adjusted for clustering using random effects only. Model 2 additionally adjusted for maternal and paternal education, asset tertiles, caste, land ownership, seasonality (cosinor terms), randomization strata, study arm and child sex as fixed effects. Model 3 additionally adjusted for height of the mother.

**Table S7.** **Coefficients of neonatal anthropometric z-scores by age at first pregnancy for length-, weight- and head circumference-for-age, and weight- for-length z scores in the first 8 days of life, showing models minimally adjusted, and adjusted with and without height of mother in the model.**

|  |  | **Model 1: Minimally adjusted (only cluster)** | | | | **Model 2: Adjusted (multiple covariates without mother's height)** | | | | **Model 3: Adjusted (multiple covariates including mother's height)** | | | |
| --- | --- | --- | --- | --- | --- | --- | --- | --- | --- | --- | --- | --- | --- |
| Outcome | Age of 1st pregnancy | n | Coeff. | (95% CI) | *p* value | n | Coeff. | (95% CI) | *p* value | n | Coeff. | (95% CI) | p value |
| Length-for-age z score <8 days | 10-14 y |  | -0.165 | (-0.657, 0.326) | 0.5098 |  | -0.166 | (-0.653, 0.322) | 0.5054 |  | 0.183 | (-0.368, 0.735) | 0.5142 |
|  | 15 y |  | -0.323 | (-0.575, -0.071) | 0.0120 |  | -0.326 | (-0.576, -0.076) | 0.0106 |  | -0.204 | (-0.493, 0.084) | 0.1646 |
|  | 16 y |  | -0.302 | (-0.5, -0.104) | 0.0029 |  | -0.299 | (-0.496, -0.102) | 0.0029 |  | -0.187 | (-0.423, 0.049) | 0.1206 |
|  | 17 y |  | -0.154 | (-0.328, 0.019) | 0.0814 |  | -0.185 | (-0.357, -0.014) | 0.0343 |  | -0.046 | (-0.246, 0.154) | 0.6522 |
|  | 18 y |  | -0.204 | (-0.415, 0.008) | 0.0588 |  | -0.257 | (-0.466, -0.048) | 0.0159 |  | -0.191 | (-0.43, 0.048) | 0.1178 |
|  | 19 y |  | -0.044 | (-0.235, 0.147) | 0.6515 |  | -0.062 | (-0.25, 0.126) | 0.5174 |  | 0.048 | (-0.166, 0.261) | 0.6611 |
|  | 20-30 y (ref) | 1364 | 0.000 |  |  | 1361 | 0.000 |  |  | 975 | 0.000 |  |  |
| Weight-for-age z score <8 days | 10-14 y |  | -0.346 | (-0.799, 0.107) | 0.1343 |  | -0.403 | (-0.852, 0.047) | 0.0794 |  | -0.169 | (-0.678, 0.341) | 0.5163 |
|  | 15 y |  | -0.332 | (-0.561, -0.103) | 0.0045 |  | -0.376 | (-0.603, -0.148) | 0.0012 |  | -0.353 | (-0.614, -0.093) | 0.0079 |
|  | 16 y |  | -0.325 | (-0.507, -0.144) | 0.0004 |  | -0.371 | (-0.552, -0.191) | 0.0001 |  | -0.346 | (-0.561, -0.131) | 0.0016 |
|  | 17 y |  | -0.264 | (-0.423, -0.106) | 0.0011 |  | -0.309 | (-0.467, -0.152) | 0.0001 |  | -0.238 | (-0.421, -0.055) | 0.0107 |
|  | 18 y |  | -0.236 | (-0.43, -0.043) | 0.0166 |  | -0.293 | (-0.484, -0.101) | 0.0028 |  | -0.241 | (-0.46, -0.022) | 0.0309 |
|  | 19 y |  | -0.190 | (-0.364, -0.015) | 0.0329 |  | -0.218 | (-0.39, -0.046) | 0.0131 |  | -0.201 | (-0.397, -0.006) | 0.0432 |
|  | 20-30 y (ref) | 1387 | 0.000 |  |  | 1384 | 0.000 |  |  | 994 | 0.000 |  |  |
| Weight-for-length z score <8 days | 10-14 y |  | -0.357 | (-0.962, 0.248) | 0.2475 |  | -0.550 | (-1.142, 0.043) | 0.0693 |  | -0.547 | (-1.255, 0.16) | 0.1296 |
|  | 15 y |  | -0.205 | (-0.509, 0.099) | 0.1861 |  | -0.310 | (-0.607, -0.013) | 0.0405 |  | -0.424 | (-0.779, -0.07) | 0.0190 |
|  | 16 y |  | -0.118 | (-0.365, 0.128) | 0.3460 |  | -0.218 | (-0.46, 0.024) | 0.0773 |  | -0.300 | (-0.595, -0.004) | 0.0468 |
|  | 17 y |  | -0.179 | (-0.387, 0.029) | 0.0910 |  | -0.230 | (-0.432, -0.027) | 0.0262 |  | -0.312 | (-0.555, -0.069) | 0.0119 |
|  | 18 y |  | -0.136 | (-0.396, 0.125) | 0.3067 |  | -0.200 | (-0.455, 0.054) | 0.1234 |  | -0.175 | (-0.476, 0.126) | 0.2551 |
|  | 19 y |  | -0.339 | (-0.568, -0.11) | 0.0038 |  | -0.391 | (-0.614, -0.167) | 0.0006 |  | -0.461 | (-0.723, -0.2) | 0.0005 |
|  | 20-30 y (ref) | 1192 | 0.000 |  |  | 1189 | 0.000 |  |  | 851 | 0.000 |  |  |
| Head circum-ference-for-age z score <8 days | 10-14 y |  | -0.561 | (-1.073, -0.049) | 0.0319 |  | -0.602 | (-1.115, -0.09) | 0.0213 |  | -0.339 | (-0.931, 0.253) | 0.2622 |
|  | 15 y |  | -0.224 | (-0.485, 0.038) | 0.0936 |  | -0.257 | (-0.519, 0.004) | 0.0540 |  | -0.312 | (-0.62, -0.005) | 0.0464 |
|  | 16 y |  | -0.309 | (-0.517, -0.102) | 0.0035 |  | -0.338 | (-0.546, -0.13) | 0.0015 |  | -0.261 | (-0.515, -0.007) | 0.0441 |
|  | 17 y |  | -0.272 | (-0.452, -0.091) | 0.0032 |  | -0.309 | (-0.489, -0.128) | 0.0008 |  | -0.245 | (-0.459, -0.03) | 0.0255 |
|  | 18 y |  | -0.185 | (-0.405, 0.034) | 0.0982 |  | -0.241 | (-0.46, -0.022) | 0.0314 |  | -0.196 | (-0.452, 0.061) | 0.1343 |
|  | 19 y |  | -0.203 | (-0.401, -0.004) | 0.0452 |  | -0.228 | (-0.425, -0.031) | 0.0236 |  | -0.203 | (-0.431, 0.026) | 0.0828 |
|  | 20-30 y (ref) | 1364 | 0.000 |  |  | 1361 | 0.000 |  |  | 975 | 0.000 |  |  |

Legend: Model 1 minimally adjusted for clustering using random effects only. Model 2 additionally adjusted for maternal and paternal education, asset tertiles, caste, land ownership, seasonality (cosinor terms), randomization strata, study arm and child sex as fixed effects. Model 3 additionally adjusted for height of the mother. Grey shaded cells in the *p* value column indicate p<0.05.

**Table S8. Coefficients from minimally and fully adjusted mixed linear regression models of neonatal anthropometric z-scores by age at marriage and pregnancy mutually adjusted for length-, weight- and head circumference-for-age, and weight- for-length z scores in the first 8 days of life, showing models minimally adjusted, and adjusted with and without height of mother in the model.** Coefficients for minimally adjusted and fully adjusted without maternal height plotted in Figure 3**.**

|  |  |  | **Model 1: Minimally adjusted (only cluster)** | | | | **Model 2: Adjusted (multiple covariates without mother's height)** | | | | **Model 3: Adjusted (multiple covariates including mother's height)** | | | |
| --- | --- | --- | --- | --- | --- | --- | --- | --- | --- | --- | --- | --- | --- | --- |
| Outcome | Exposure | Age group | n | Coeff. | (95% CI) | *p* value | n | Coeff. | (95% CI) | *p* value | n | Coeff. | (95% CI) | p value |
| Length-for-age z score <8 days | Age married | 10-13 y |  | -0.064 | (-0.374, 0.245) | 0.6837 |  | 0.035 | (-0.275, 0.346) | 0.8232 |  | -0.049 | (-0.397, 0.299) | 0.7838 |
|  |  | 14 y |  | 0.049 | (-0.186, 0.283) | 0.6843 |  | 0.140 | (-0.096, 0.376) | 0.2445 |  | 0.209 | (-0.057, 0.474) | 0.1233 |
|  |  | 15 y |  | 0.021 | (-0.204, 0.246) | 0.8553 |  | 0.056 | (-0.168, 0.281) | 0.6230 |  | 0.056 | (-0.2, 0.313) | 0.6668 |
|  |  | 16 y |  | 0.063 | (-0.16, 0.286) | 0.5810 |  | 0.093 | (-0.129, 0.314) | 0.4110 |  | 0.049 | (-0.203, 0.301) | 0.7022 |
|  |  | 17 y |  | 0.127 | (-0.093, 0.348) | 0.2588 |  | 0.133 | (-0.084, 0.351) | 0.2289 |  | 0.091 | (-0.153, 0.336) | 0.4624 |
|  |  | 18-24 y (ref) | 1346 | 0.000 |  |  | 1345 | 0 |  |  | 961 | 0 |  |  |
|  | Age at 1st pregnancy | 10-14 y |  | -0.106 | (-0.626, 0.413) | 0.6883 |  | -0.167 | (-0.682, 0.349) | 0.5262 |  | 0.197 | (-0.378, 0.772) | 0.5023 |
|  |  | 15 y |  | -0.320 | (-0.59, -0.049) | 0.0205 |  | -0.356 | (-0.625, -0.087) | 0.0096 |  | -0.253 | (-0.562, 0.056) | 0.1080 |
|  |  | 16 y |  | -0.302 | (-0.519, -0.084) | 0.0066 |  | -0.313 | (-0.53, -0.097) | 0.0046 |  | -0.192 | (-0.448, 0.065) | 0.1439 |
|  |  | 17 y |  | -0.181 | (-0.372, 0.01) | 0.0637 |  | -0.217 | (-0.407, -0.028) | 0.0245 |  | -0.050 | (-0.268, 0.169) | 0.6546 |
|  |  | 18 y |  | -0.247 | (-0.467, -0.027) | 0.0280 |  | -0.295 | (-0.512, -0.077) | 0.0080 |  | -0.209 | (-0.456, 0.038) | 0.0980 |
|  |  | 19 y |  | -0.059 | (-0.252, 0.135) | 0.5526 |  | -0.063 | (-0.253, 0.127) | 0.5177 |  | 0.070 | (-0.146, 0.286) | 0.5242 |
|  |  | 20-30 y (ref) |  | 0.000 |  |  |  | 0.000 |  |  |  | 0.000 |  |  |
| Weight-for-age z score <8 days | Age married | 10-13 y |  | 0.184 | (-0.101, 0.469) | 0.2056 |  | 0.391 | (0.106, 0.676) | 0.0072 |  | 0.419 | (0.099, 0.739) | 0.0103 |
|  |  | 14 y |  | 0.188 | (-0.026, 0.403) | 0.0857 |  | 0.364 | (0.149, 0.58) | 0.0009 |  | 0.361 | (0.117, 0.605) | 0.0037 |
|  |  | 15 y |  | 0.137 | (-0.068, 0.343) | 0.1906 |  | 0.247 | (0.042, 0.452) | 0.0181 |  | 0.258 | (0.024, 0.493) | 0.0309 |
|  |  | 16 y |  | 0.140 | (-0.064, 0.344) | 0.1780 |  | 0.216 | (0.014, 0.418) | 0.0359 |  | 0.212 | (-0.017, 0.442) | 0.0701 |
|  |  | 17 y |  | 0.102 | (-0.099, 0.302) | 0.3210 |  | 0.146 | (-0.051, 0.344) | 0.1471 |  | 0.138 | (-0.085, 0.361) | 0.2262 |
|  |  | 18-24 y (ref) | 1369 | 0.000 |  |  | 1368 | 0 |  |  | 980 | 0 |  |  |
|  | Age at 1st pregnancy | 10-14 y |  | -0.439 | (-0.917, 0.04) | 0.0723 |  | -0.612 | (-1.086, -0.138) | 0.0114 |  | -0.383 | (-0.913, 0.148) | 0.1577 |
|  |  | 15 y |  | -0.407 | (-0.653, -0.162) | 0.0012 |  | -0.528 | (-0.773, -0.284) | 0.0000 |  | -0.502 | (-0.781, -0.223) | 0.0004 |
|  |  | 16 y |  | -0.387 | (-0.585, -0.188) | 0.0001 |  | -0.475 | (-0.673, -0.278) | 0.0000 |  | -0.439 | (-0.672, -0.205) | 0.0002 |
|  |  | 17 y |  | -0.313 | (-0.487, -0.139) | 0.0004 |  | -0.382 | (-0.555, -0.21) | 0.0000 |  | -0.297 | (-0.496, -0.099) | 0.0033 |
|  |  | 18 y |  | -0.261 | (-0.462, -0.06) | 0.0110 |  | -0.322 | (-0.521, -0.124) | 0.0015 |  | -0.263 | (-0.488, -0.037) | 0.0223 |
|  |  | 19 y |  | -0.190 | (-0.367, -0.013) | 0.0349 |  | -0.202 | (-0.376, -0.029) | 0.0221 |  | -0.172 | (-0.369, 0.025) | 0.0875 |
|  |  | 20-30 y (ref) |  | 0.000 |  |  |  | 0.000 |  |  |  | 0.000 |  |  |
| Weight-for-length z score <8 days | Age married | 10-13 y |  | 0.110 | (-0.273, 0.493) | 0.5729 |  | 0.357 | (-0.023, 0.736) | 0.0655 |  | 0.465 | (0.029, 0.902) | 0.0367 |
|  |  | 14 y |  | 0.101 | (-0.183, 0.385) | 0.4860 |  | 0.275 | (-0.005, 0.555) | 0.0543 |  | 0.240 | (-0.082, 0.562) | 0.1441 |
|  |  | 15 y |  | 0.024 | (-0.249, 0.296) | 0.8641 |  | 0.167 | (-0.101, 0.435) | 0.2212 |  | 0.159 | (-0.156, 0.474) | 0.3228 |
|  |  | 16 y |  | -0.041 | (-0.31, 0.228) | 0.7662 |  | 0.046 | (-0.216, 0.308) | 0.7309 |  | 0.007 | (-0.301, 0.314) | 0.9662 |
|  |  | 17 y |  | -0.050 | (-0.313, 0.214) | 0.7122 |  | 0.002 | (-0.254, 0.258) | 0.9868 |  | -0.026 | (-0.321, 0.269) | 0.8631 |
|  |  | 18-24 y (ref) | 1175 | 0.000 |  |  | 1174 | 0 |  |  | 838 | 0 |  |  |
|  | Age at 1st pregnancy | 10-14 y |  | -0.448 | (-1.089, 0.193) | 0.1707 |  | -0.779 | (-1.405, -0.152) | 0.0148 |  | -0.815 | (-1.551, -0.079) | 0.0299 |
|  |  | 15 y |  | -0.261 | (-0.588, 0.067) | 0.1184 |  | -0.459 | (-0.779, -0.139) | 0.0050 |  | -0.566 | (-0.945, -0.186) | 0.0035 |
|  |  | 16 y |  | -0.143 | (-0.413, 0.128) | 0.3013 |  | -0.301 | (-0.566, -0.036) | 0.0261 |  | -0.360 | (-0.681, -0.039) | 0.0279 |
|  |  | 17 y |  | -0.159 | (-0.39, 0.072) | 0.1765 |  | -0.241 | (-0.466, -0.017) | 0.0350 |  | -0.302 | (-0.568, -0.035) | 0.0264 |
|  |  | 18 y |  | -0.101 | (-0.373, 0.171) | 0.4679 |  | -0.176 | (-0.441, 0.089) | 0.1935 |  | -0.131 | (-0.441, 0.18) | 0.4091 |
|  |  | 19 y |  | -0.331 | (-0.564, -0.097) | 0.0055 |  | -0.372 | (-0.597, -0.146) | 0.0013 |  | -0.439 | (-0.704, -0.174) | 0.0011 |
|  |  | 20-30 y (ref) |  | 0.000 |  |  |  | 0.000 |  |  |  | 0.000 |  |  |
| Head circumference-for-age z score <8 days | Age married | 10-13 y |  | 0.041 | (-0.283, 0.364) | 0.8056 |  | 0.165 | (-0.163, 0.494) | 0.3230 |  | 0.214 | (-0.162, 0.59) | 0.2655 |
|  |  | 14 y |  | 0.066 | (-0.179, 0.311) | 0.5953 |  | 0.181 | (-0.067, 0.43) | 0.1529 |  | 0.250 | (-0.036, 0.537) | 0.0869 |
|  |  | 15 y |  | -0.017 | (-0.251, 0.218) | 0.8881 |  | 0.064 | (-0.172, 0.3) | 0.5966 |  | 0.177 | (-0.099, 0.452) | 0.2092 |
|  |  | 16 y |  | 0.096 | (-0.137, 0.329) | 0.4189 |  | 0.139 | (-0.094, 0.371) | 0.2437 |  | 0.165 | (-0.106, 0.435) | 0.2336 |
|  |  | 17 y |  | 0.138 | (-0.09, 0.366) | 0.2356 |  | 0.162 | (-0.065, 0.389) | 0.1623 |  | 0.241 | (-0.02, 0.503) | 0.0704 |
|  |  | 18-24 y (ref) | 1347 | 0.000 |  |  | 1346 | 0 |  |  | 962 | 0 |  |  |
|  | Age at 1st pregnancy | 10-14 y |  | -0.561 | (-1.102, -0.021) | 0.0418 |  | -0.676 | (-1.218, -0.135) | 0.0144 |  | -0.423 | (-1.041, 0.196) | 0.1804 |
|  |  | 15 y |  | -0.215 | (-0.495, 0.066) | 0.1333 |  | -0.300 | (-0.581, -0.018) | 0.0370 |  | -0.393 | (-0.722, -0.063) | 0.0195 |
|  |  | 16 y |  | -0.304 | (-0.531, -0.077) | 0.0088 |  | -0.362 | (-0.59, -0.134) | 0.0019 |  | -0.313 | (-0.59, -0.036) | 0.0266 |
|  |  | 17 y |  | -0.312 | (-0.511, -0.114) | 0.0020 |  | -0.361 | (-0.56, -0.163) | 0.0004 |  | -0.308 | (-0.542, -0.074) | 0.0100 |
|  |  | 18 y |  | -0.233 | (-0.461, -0.004) | 0.0458 |  | -0.287 | (-0.515, -0.06) | 0.0134 |  | -0.249 | (-0.513, 0.015) | 0.0650 |
|  |  | 19 y |  | -0.202 | (-0.402, -0.001) | 0.0490 |  | -0.219 | (-0.419, -0.02) | 0.0308 |  | -0.178 | (-0.409, 0.054) | 0.1326 |
|  |  | 20-30 y (ref) |  | 0.000 |  |  |  | 0.000 |  |  |  | 0.000 |  |  |

Legend: Model 1 minimally adjusted for clustering using random effects only. Model 2 additionally adjusted for maternal and paternal education, asset tertiles, caste, land ownership, seasonality (cosinor terms), randomization strata, study arm and child sex as fixed effects. Model 3 additionally adjusted for height of the mother. Grey shaded cells in the *p* value column indicate p<0.05.

**Table S9. Odds ratios from minimally and fully adjusted models of neonatal anthropometric stunting and wasting by age at marriage and pregnancy alone and mutually adjusted scores in the first 8 days of life, showing models minimally adjusted, and adjusted with and without height of mother in the model.** Odds ratios plotted in Figure 4.

|  |  |  | **Model 1: Minimally adjusted (only cluster)** | | | | **Model 2: Adjusted (multiple covariates without mother's height)** | | | | **Model 3: Adjusted (multiple covariates including mother's height)** | | | |
| --- | --- | --- | --- | --- | --- | --- | --- | --- | --- | --- | --- | --- | --- | --- |
| **Outcome** | **Exposure** | **Age group** | **n** | **OR** | **(95% CI)** | ***p* value** | **n** | **OR** | **(95% CI)** | ***p* value** | **n** | **OR** | **(95% CI)** | **p value** |
| Stunting <8 days | Age married | 10-13 y |  | 2.191 | (1.147, 4.186) | 0.0175 |  | 1.935 | (0.992, 3.773) | 0.0527 |  | 1.969 | (0.887, 4.374) | 0.0960 |
|  |  | 14 y |  | 2.122 | (1.272, 3.539) | 0.0039 |  | 1.862 | (1.099, 3.156) | 0.0209 |  | 1.615 | (0.859, 3.034) | 0.1365 |
|  |  | 15 y |  | 2.006 | (1.241, 3.242) | 0.0045 |  | 1.931 | (1.179, 3.162) | 0.0090 |  | 1.844 | (1.012, 3.361) | 0.0456 |
|  |  | 16 y |  | 1.526 | (0.937, 2.486) | 0.0894 |  | 1.471 | (0.892, 2.425) | 0.1304 |  | 1.634 | (0.898, 2.975) | 0.1082 |
|  |  | 17 y |  | 1.440 | (0.863, 2.401) | 0.1628 |  | 1.420 | (0.842, 2.398) | 0.1888 |  | 1.493 | (0.8, 2.786) | 0.2076 |
|  |  | 18-24 y (ref) | 1346 | 1 |  |  | 1345 | 1.000 |  |  | 961 | 1.000 |  |  |
| Wasting <8 days | Age married | 10-13 y |  | 1.243 | (0.584, 2.643) | 0.5726 |  | 1.055 | (0.478, 2.33) | 0.8948 |  | 0.830 | (0.28, 2.462) | 0.7373 |
|  |  | 14 y |  | 0.977 | (0.548, 1.742) | 0.9382 |  | 0.889 | (0.486, 1.625) | 0.7019 |  | 1.455 | (0.706, 2.999) | 0.3096 |
|  |  | 15 y |  | 0.922 | (0.536, 1.587) | 0.7705 |  | 0.890 | (0.507, 1.562) | 0.6839 |  | 1.530 | (0.755, 3.097) | 0.2376 |
|  |  | 16 y |  | 1.016 | (0.598, 1.726) | 0.9545 |  | 1.016 | (0.588, 1.755) | 0.9550 |  | 1.372 | (0.688, 2.739) | 0.3692 |
|  |  | 17 y |  | 1.284 | (0.748, 2.205) | 0.3649 |  | 1.354 | (0.774, 2.368) | 0.2889 |  | 1.961 | (0.97, 3.961) | 0.0606 |
|  |  | 18-24 y (ref) | 1175 | 1 |  |  | 1174 | 1.000 |  |  | 838 | 1.000 |  |  |
| Stunting <8 days | Age of 1st pregnancy | 10-14 y |  | 1.484 | (0.514, 4.288) | 0.4659 |  | 1.557 | (0.52, 4.665) | 0.4291 |  | 0.454 | (0.106, 1.942) | 0.2869 |
|  |  | 15 y |  | 1.623 | (0.931, 2.831) | 0.0877 |  | 1.772 | (0.997, 3.149) | 0.0514 |  | 1.400 | (0.713, 2.749) | 0.3284 |
|  |  | 16 y |  | 1.790 | (1.15, 2.787) | 0.0099 |  | 1.894 | (1.195, 3.003) | 0.0066 |  | 1.209 | (0.686, 2.131) | 0.5121 |
|  |  | 17 y |  | 0.988 | (0.656, 1.488) | 0.9543 |  | 1.054 | (0.689, 1.611) | 0.8087 |  | 0.694 | (0.417, 1.154) | 0.1594 |
|  |  | 18 y |  | 1.431 | (0.888, 2.306) | 0.1412 |  | 1.631 | (0.995, 2.674) | 0.0521 |  | 1.354 | (0.761, 2.406) | 0.3024 |
|  |  | 19 y |  | 0.955 | (0.605, 1.507) | 0.8431 |  | 1.016 | (0.636, 1.623) | 0.9468 |  | 0.732 | (0.421, 1.275) | 0.2711 |
|  |  | 20-30 y (ref) | 1364 | 1 |  |  | 1361 | 1.000 |  |  | 975 | 1.000 |  |  |
| Wasting <8 days | Age of 1st pregnancy | 10-14 y |  | 1.063 | (0.28, 4.038) | 0.9284 |  | 1.382 | (0.352, 5.422) | 0.6431 |  | 1.866 | (0.344, 10.131) | 0.4697 |
|  |  | 15 y |  | 0.921 | (0.453, 1.875) | 0.8215 |  | 1.022 | (0.489, 2.135) | 0.9547 |  | 1.054 | (0.424, 2.617) | 0.9104 |
|  |  | 16 y |  | 0.994 | (0.562, 1.755) | 0.9821 |  | 1.222 | (0.678, 2.203) | 0.5052 |  | 1.755 | (0.856, 3.596) | 0.1245 |
|  |  | 17 y |  | 1.163 | (0.725, 1.865) | 0.5317 |  | 1.243 | (0.763, 2.023) | 0.3823 |  | 1.705 | (0.938, 3.101) | 0.0803 |
|  |  | 18 y |  | 1.357 | (0.766, 2.403) | 0.2955 |  | 1.535 | (0.849, 2.774) | 0.1561 |  | 1.889 | (0.92, 3.878) | 0.0831 |
|  |  | 19 y |  | 1.289 | (0.774, 2.148) | 0.3297 |  | 1.359 | (0.804, 2.297) | 0.2524 |  | 1.333 | (0.693, 2.563) | 0.3896 |
|  |  | 20-30 y (ref) | 1192 | 1.000 |  |  | 1189 | 1.000 |  |  | 851 | 1.000 |  |  |
| Stunting <8 days | Age married | 10-13 y |  | 2.074 | (1.033, 4.166) | 0.0403 |  | 1.744 | (0.845, 3.601) | 0.1326 |  | 2.299 | (0.977, 5.41) | 0.0567 |
|  |  | 14 y |  | 1.914 | (1.101, 3.328) | 0.0214 |  | 1.607 | (0.906, 2.848) | 0.1047 |  | 1.614 | (0.815, 3.199) | 0.1699 |
|  |  | 15 y |  | 1.770 | (1.032, 3.037) | 0.0381 |  | 1.634 | (0.937, 2.85) | 0.0837 |  | 1.920 | (0.982, 3.754) | 0.0565 |
|  |  | 16 y |  | 1.471 | (0.854, 2.534) | 0.1640 |  | 1.371 | (0.785, 2.396) | 0.2676 |  | 1.895 | (0.973, 3.69) | 0.0603 |
|  |  | 17 y |  | 1.408 | (0.821, 2.413) | 0.2137 |  | 1.345 | (0.774, 2.335) | 0.2927 |  | 1.623 | (0.844, 3.124) | 0.1468 |
|  |  | 18-24 y (ref) | 1346 | 1.000 |  |  | 1345 | 1.000 |  |  | 961 | 1.000 |  |  |
|  | Age at 1st pregnancy | 10-14 y |  | 1.051 | (0.345, 3.206) | 0.9299 |  | 1.223 | (0.385, 3.882) | 0.7332 |  | 0.334 | (0.074, 1.508) | 0.1538 |
|  |  | 15 y |  | 1.270 | (0.703, 2.292) | 0.4280 |  | 1.460 | (0.792, 2.694) | 0.2256 |  | 1.173 | (0.57, 2.418) | 0.6646 |
|  |  | 16 y |  | 1.510 | (0.934, 2.443) | 0.0926 |  | 1.643 | (0.998, 2.706) | 0.0509 |  | 0.977 | (0.529, 1.806) | 0.9411 |
|  |  | 17 y |  | 0.891 | (0.571, 1.389) | 0.6099 |  | 0.955 | (0.604, 1.512) | 0.8446 |  | 0.558 | (0.322, 0.969) | 0.0381 |
|  |  | 18 y |  | 1.399 | (0.851, 2.299) | 0.1850 |  | 1.583 | (0.949, 2.64) | 0.0787 |  | 1.242 | (0.685, 2.255) | 0.4751 |
|  |  | 19 y |  | 1.004 | (0.632, 1.595) | 0.9869 |  | 1.048 | (0.652, 1.684) | 0.8473 |  | 0.744 | (0.422, 1.309) | 0.3045 |
|  |  | 20-30 y (ref) |  | 1.000 |  |  |  | 1.000 |  |  |  | 1.000 |  |  |
| Wasting <8 days | Age married | 10-13 y |  | 1.353 | (0.599, 3.057) | 0.4668 |  | 1.004 | (0.421, 2.393) | 0.9927 |  | 0.698 | (0.218, 2.24) | 0.5460 |
|  |  | 14 y |  | 1.061 | (0.564, 1.996) | 0.8548 |  | 0.851 | (0.436, 1.66) | 0.6360 |  | 1.326 | (0.595, 2.957) | 0.4898 |
|  |  | 15 y |  | 0.971 | (0.525, 1.793) | 0.9242 |  | 0.822 | (0.432, 1.565) | 0.5511 |  | 1.277 | (0.576, 2.83) | 0.5479 |
|  |  | 16 y |  | 1.028 | (0.565, 1.871) | 0.9290 |  | 0.943 | (0.506, 1.755) | 0.8525 |  | 1.070 | (0.488, 2.348) | 0.8655 |
|  |  | 17 y |  | 1.229 | (0.694, 2.179) | 0.4795 |  | 1.241 | (0.687, 2.244) | 0.4745 |  | 1.676 | (0.805, 3.487) | 0.1675 |
|  |  | 18-24 y (ref) | 1175 | 1.000 |  |  | 1174 | 1.000 |  |  | 838 | 1.000 |  |  |
|  | Age at 1st pregnancy | 10-14 y |  | 0.915 | (0.225, 3.715) | 0.9014 |  | 1.400 | (0.328, 5.982) | 0.6498 |  | 2.203 | (0.374, 12.983) | 0.3826 |
|  |  | 15 y |  | 0.940 | (0.441, 2.003) | 0.8731 |  | 1.175 | (0.532, 2.596) | 0.6906 |  | 1.042 | (0.397, 2.735) | 0.9341 |
|  |  | 16 y |  | 1.056 | (0.57, 1.955) | 0.8634 |  | 1.406 | (0.738, 2.677) | 0.3002 |  | 1.770 | (0.811, 3.861) | 0.1517 |
|  |  | 17 y |  | 1.127 | (0.67, 1.893) | 0.6525 |  | 1.230 | (0.718, 2.106) | 0.4510 |  | 1.613 | (0.836, 3.11) | 0.1539 |
|  |  | 18 y |  | 1.335 | (0.737, 2.419) | 0.3398 |  | 1.498 | (0.809, 2.773) | 0.1983 |  | 1.816 | (0.867, 3.804) | 0.1138 |
|  |  | 19 y |  | 1.301 | (0.775, 2.183) | 0.3198 |  | 1.330 | (0.781, 2.264) | 0.2943 |  | 1.333 | (0.684, 2.6) | 0.3987 |
|  |  | 20-30 y (ref) |  | 1.000 |  |  |  | 1.000 |  |  |  | 1.000 |  |  |

Legend: Model 1 minimally adjusted for clustering using random effects only. Model 2 additionally adjusted for maternal and paternal education, asset tertiles, caste, land ownership, seasonality (cosinor terms), randomization strata, study arm and child sex as fixed effects. Model 3 additionally adjusted for height of the mother. Grey shaded cells in the *p* value column indicate p<0.05.

**Table S10.** **Coefficients from minimally and fully adjusted mixed linear regression models of infant anthropometric *z* scores by age at marriage for length-, weight- and head circumference-for-age, and weight- for-length z scores from 180 to 365 days, showing models minimally adjusted, and adjusted with and without height of mother in the model.**

|  |  | **Model 1: Minimally adjusted (only cluster and child age)** | | | | **Model 2: Adjusted (multiple covariates without mother's height)** | | | | **Model 3: Adjusted (multiple covariates including mother's height)** | | | |
| --- | --- | --- | --- | --- | --- | --- | --- | --- | --- | --- | --- | --- | --- |
| **Outcome** | **Age married** | **n** | **Coeff.** | **(95% CI)** | ***p* value** | **n** | **Coeff.** | **(95% CI)** | ***p* value** | **n** | **Coeff.** | **(95% CI)** | ***p* value** |
| Length-for-age z score 6 to 12m | 10-13 y |  | -0.390 | (-0.635, -0.144) | 0.0018 |  | -0.302 | (-0.545, -0.058) | 0.0152 |  | -0.252 | (-0.504, 0.001) | 0.0509 |
|  | 14 y |  | -0.167 | (-0.341, 0.006) | 0.0587 |  | -0.095 | (-0.268, 0.078) | 0.2831 |  | -0.070 | (-0.25, 0.109) | 0.4438 |
|  | 15 y |  | -0.187 | (-0.344, -0.03) | 0.0197 |  | -0.131 | (-0.287, 0.025) | 0.1000 |  | -0.139 | (-0.302, 0.024) | 0.0942 |
|  | 16 y |  | -0.159 | (-0.319, 0.002) | 0.0526 |  | -0.125 | (-0.284, 0.034) | 0.1223 |  | -0.154 | (-0.319, 0.011) | 0.0679 |
|  | 17 y |  | -0.158 | (-0.326, 0.01) | 0.0648 |  | -0.142 | (-0.306, 0.023) | 0.0924 |  | -0.142 | (-0.313, 0.029) | 0.1042 |
|  | 18-24 y (ref) | 2068 | 0.000 |  |  | 2068 | 0.000 |  |  | 1726 | 0.000 |  |  |
| Weight-for-age z score 6 to 12m | 10-13 y |  | -0.181 | (-0.413, 0.05) | 0.1248 |  | -0.113 | (-0.343, 0.118) | 0.3381 |  | -0.041 | (-0.282, 0.201) | 0.7406 |
|  | 14 y |  | -0.047 | (-0.21, 0.117) | 0.5751 |  | 0.005 | (-0.158, 0.169) | 0.9495 |  | 0.032 | (-0.139, 0.204) | 0.7111 |
|  | 15 y |  | -0.022 | (-0.171, 0.126) | 0.7690 |  | 0.000 | (-0.148, 0.148) | 0.9987 |  | -0.010 | (-0.167, 0.146) | 0.8957 |
|  | 16 y |  | -0.007 | (-0.158, 0.145) | 0.9303 |  | -0.014 | (-0.164, 0.136) | 0.8558 |  | -0.046 | (-0.204, 0.112) | 0.5711 |
|  | 17 y |  | -0.018 | (-0.176, 0.14) | 0.8219 |  | -0.035 | (-0.191, 0.121) | 0.6581 |  | -0.011 | (-0.175, 0.152) | 0.8919 |
|  | 18-24 y (ref) | 2048 | 0.000 |  |  | 2047 | 0.000 |  |  | 1708 | 0.000 |  |  |
| Weight-for-length z score 6 to 12m | 10-13 y |  | 0.081 | (-0.134, 0.297) | 0.4601 |  | 0.115 | (-0.102, 0.331) | 0.2987 |  | 0.183 | (-0.052, 0.418) | 0.1275 |
|  | 14 y |  | 0.051 | (-0.1, 0.202) | 0.5094 |  | 0.079 | (-0.073, 0.232) | 0.3087 |  | 0.106 | (-0.059, 0.271) | 0.2082 |
|  | 15 y |  | 0.118 | (-0.019, 0.256) | 0.0920 |  | 0.124 | (-0.014, 0.262) | 0.0782 |  | 0.122 | (-0.028, 0.273) | 0.1120 |
|  | 16 y |  | 0.072 | (-0.068, 0.212) | 0.3139 |  | 0.054 | (-0.086, 0.194) | 0.4520 |  | 0.039 | (-0.114, 0.192) | 0.6167 |
|  | 17 y |  | 0.102 | (-0.044, 0.248) | 0.1715 |  | 0.083 | (-0.062, 0.229) | 0.2629 |  | 0.123 | (-0.036, 0.281) | 0.1298 |
|  | 18-24 y (ref) | 2042 | 0.000 |  |  | 2041 | 0.000 |  |  | 1703 | 0.000 |  |  |
| Head circumference-for-age z score 6 to 12m | 10-13 y |  | -0.121 | (-0.342, 0.101) | 0.2855 |  | -0.032 | (-0.252, 0.189) | 0.7776 |  | -0.055 | (-0.288, 0.177) | 0.6416 |
|  | 14 y |  | -0.004 | (-0.163, 0.154) | 0.9590 |  | 0.056 | (-0.102, 0.215) | 0.4853 |  | 0.076 | (-0.092, 0.243) | 0.3758 |
|  | 15 y |  | -0.019 | (-0.162, 0.124) | 0.7989 |  | 0.035 | (-0.108, 0.177) | 0.6320 |  | 0.016 | (-0.135, 0.167) | 0.8327 |
|  | 16 y |  | 0.012 | (-0.133, 0.158) | 0.8693 |  | 0.039 | (-0.105, 0.183) | 0.5998 |  | -0.017 | (-0.169, 0.136) | 0.8317 |
|  | 17 y |  | -0.008 | (-0.159, 0.143) | 0.9165 |  | 0.007 | (-0.142, 0.156) | 0.9290 |  | 0.047 | (-0.111, 0.205) | 0.5629 |
|  | 18-24 y (ref) | 2064 | 0.000 |  |  | 2064 | 0.000 |  |  | 1721 | 0.000 |  |  |

Legend: Model 1 minimally adjusted for clustering using random effects and child age as a fixed effect. Model 2 additionally adjusted for maternal and paternal education, asset tertiles, caste, land ownership, seasonality (cosinor terms), randomization strata, study arm and child sex as fixed effects. Model 3 additionally adjusted for height of the mother. Grey shaded cells in the *p* value column indicate p<0.05.

**Table S11.** **Coefficients from mixed linear regressions of infant anthropometric z scores by age at first pregnancy for length-, weight- and head circumference-for-age, and weight-for-length z scores from 180 to 365 days, showing models minimally adjusted, and adjusted with and without height of mother in the model.**

|  |  | **Model 1: Minimally adjusted (only cluster and child age)** | | | | **Model 2: Adjusted (multiple covariates without mother's height)** | | | | **Model 3: Adjusted (multiple covariates including mother's height)** | | | |
| --- | --- | --- | --- | --- | --- | --- | --- | --- | --- | --- | --- | --- | --- |
| **Outcome** | **Age of 1st pregnancy** | **n** | **Coeff.** | **(95% CI)** | ***p* value** | **n** | **Coeff.** | **(95% CI)** | ***p* value** | **n** | **Coeff.** | **(95% CI)** | **p value** |
| Length-for-age z score 6 to 12m | 10-14 y |  | -0.394 | (-0.812, 0.023) | 0.0641 |  | -0.381 | (-0.793, 0.032) | 0.0706 |  | -0.100 | (-0.549, 0.349) | 0.6621 |
|  | 15 y |  | -0.212 | (-0.403, -0.021) | 0.0299 |  | -0.170 | (-0.36, 0.019) | 0.0773 |  | -0.099 | (-0.296, 0.098) | 0.3257 |
|  | 16 y |  | -0.208 | (-0.365, -0.052) | 0.0091 |  | -0.177 | (-0.332, -0.021) | 0.0264 |  | -0.108 | (-0.274, 0.058) | 0.2016 |
|  | 17 y |  | -0.226 | (-0.36, -0.093) | 0.0009 |  | -0.212 | (-0.345, -0.079) | 0.0018 |  | -0.175 | (-0.313, -0.038) | 0.0126 |
|  | 18 y |  | -0.224 | (-0.388, -0.061) | 0.0073 |  | -0.219 | (-0.381, -0.056) | 0.0083 |  | -0.219 | (-0.387, -0.052) | 0.0103 |
|  | 19 y |  | -0.103 | (-0.255, 0.048) | 0.1810 |  | -0.081 | (-0.231, 0.07) | 0.2930 |  | -0.060 | (-0.214, 0.094) | 0.4437 |
|  | 20-30 y (ref) | 2281 | 0.000 |  |  | 2265 | 0.000 |  |  | 1881 | 0.000 |  |  |
| Weight-for-age z score 6 to 12m | 10-14 y |  | -0.386 | (-0.773, 0.001) | 0.0505 |  | -0.353 | (-0.736, 0.031) | 0.0714 |  | -0.061 | (-0.48, 0.358) | 0.7757 |
|  | 15 y |  | -0.061 | (-0.241, 0.119) | 0.5056 |  | -0.029 | (-0.208, 0.149) | 0.7465 |  | 0.037 | (-0.152, 0.225) | 0.7027 |
|  | 16 y |  | -0.059 | (-0.207, 0.089) | 0.4328 |  | -0.045 | (-0.192, 0.103) | 0.5532 |  | -0.017 | (-0.175, 0.142) | 0.8378 |
|  | 17 y |  | -0.053 | (-0.179, 0.073) | 0.4105 |  | -0.058 | (-0.184, 0.069) | 0.3699 |  | -0.035 | (-0.167, 0.098) | 0.6089 |
|  | 18 y |  | 0.025 | (-0.129, 0.18) | 0.7486 |  | 0.013 | (-0.141, 0.166) | 0.8714 |  | -0.010 | (-0.17, 0.15) | 0.9029 |
|  | 19 y |  | -0.034 | (-0.178, 0.109) | 0.6417 |  | -0.030 | (-0.173, 0.112) | 0.6769 |  | -0.020 | (-0.168, 0.127) | 0.7864 |
|  | 20-30 y (ref) | 2262 | 0.000 |  |  | 2243 | 0.000 |  |  | 1862 | 0.000 |  |  |
| Weight-for-length z score 6 to 12m | 10-14 y |  | -0.183 | (-0.547, 0.18) | 0.3222 |  | -0.165 | (-0.528, 0.198) | 0.3741 |  | 0.022 | (-0.393, 0.437) | 0.9179 |
|  | 15 y |  | 0.064 | (-0.103, 0.231) | 0.4525 |  | 0.082 | (-0.085, 0.249) | 0.3359 |  | 0.093 | (-0.089, 0.276) | 0.3172 |
|  | 16 y |  | 0.049 | (-0.088, 0.187) | 0.4796 |  | 0.050 | (-0.088, 0.188) | 0.4760 |  | 0.016 | (-0.137, 0.17) | 0.8331 |
|  | 17 y |  | 0.090 | (-0.027, 0.207) | 0.1321 |  | 0.081 | (-0.037, 0.199) | 0.1786 |  | 0.071 | (-0.057, 0.199) | 0.2767 |
|  | 18 y |  | 0.193 | (0.05, 0.336) | 0.0081 |  | 0.179 | (0.036, 0.323) | 0.0141 |  | 0.140 | (-0.015, 0.296) | 0.0770 |
|  | 19 y |  | 0.044 | (-0.089, 0.177) | 0.5191 |  | 0.036 | (-0.097, 0.169) | 0.5977 |  | 0.009 | (-0.134, 0.151) | 0.9050 |
|  | 20-30 y (ref) | 2252 | 0.000 |  |  | 2235 | 0.000 |  |  | 1855 | 0.000 |  |  |
| Head circumference-for-age z score 6 to 12m | 10-14 y |  | -0.387 | (-0.758, -0.015) | 0.0414 |  | -0.339 | (-0.708, 0.029) | 0.0711 |  | -0.183 | (-0.591, 0.226) | 0.3808 |
|  | 15 y |  | -0.068 | (-0.241, 0.106) | 0.4428 |  | -0.038 | (-0.21, 0.134) | 0.6666 |  | 0.003 | (-0.181, 0.187) | 0.9730 |
|  | 16 y |  | -0.130 | (-0.273, 0.012) | 0.0730 |  | -0.106 | (-0.248, 0.036) | 0.1428 |  | -0.072 | (-0.226, 0.082) | 0.3595 |
|  | 17 y |  | -0.088 | (-0.209, 0.033) | 0.1556 |  | -0.083 | (-0.204, 0.038) | 0.1795 |  | -0.060 | (-0.188, 0.069) | 0.3625 |
|  | 18 y |  | -0.077 | (-0.226, 0.072) | 0.3131 |  | -0.073 | (-0.221, 0.075) | 0.3344 |  | -0.098 | (-0.254, 0.059) | 0.2214 |
|  | 19 y |  | -0.058 | (-0.196, 0.079) | 0.4049 |  | -0.049 | (-0.186, 0.088) | 0.4808 |  | -0.041 | (-0.184, 0.103) | 0.5788 |
|  | 20-30 y (ref) | 2277 | 0.000 |  |  | 2260 | 0.000 |  |  | 1875 | 0.000 |  |  |

Legend: Model 1 minimally adjusted for clustering using random effects and child age as a fixed effect. Model 2 additionally adjusted for maternal and paternal education, asset tertiles, caste, land ownership, seasonality (cosinor terms), randomization strata, study arm and child sex as fixed effects. Model 3 additionally adjusted for height of the mother. Grey shaded cells in the *p* value column indicate p<0.05.

**Table S12.** **Coefficients from mixed linear regression models for anthropometric z-scores for length-, weight- and head- circumference-for-age and weight-for-length from 180 to 365 days by age of marriage and age of first pregnancy mutually adjusted, showing models minimally adjusted, adjusted with and without height of mother in the model.** Coefficients plotted in Figure 5.

|  |  |  | **Model 1: Minimally adjusted (only cluster and child age)** | | | | **Model 2: Adjusted (multiple covariates without mother's height)** | | | | **Model 3: Adjusted (multiple covariates including mother's height)** | | | |
| --- | --- | --- | --- | --- | --- | --- | --- | --- | --- | --- | --- | --- | --- | --- |
| **Outcome** | **Exposure** | **Age group** | **n** | **Coeff.** | **(95% CI)** | ***p value*** | **n** | **Coeff.** | **(95% CI)** | ***p value*** | **n** | **Coeff.** | **(95% CI)** | ***p value*** |
| Length-for-age z score 6 to 12m | Age married | 10-13 y |  | -0.272 | (-0.532, -0.012) | 0.0400 |  | -0.171 | (-0.43, 0.088) | 0.1966 |  | -0.192 | (-0.459, 0.074) | 0.1565 |
|  |  | 14 y |  | -0.070 | (-0.257, 0.118) | 0.4676 |  | 0.012 | (-0.178, 0.201) | 0.9051 |  | -0.016 | (-0.212, 0.18) | 0.8718 |
|  |  | 15 y |  | -0.079 | (-0.255, 0.096) | 0.3766 |  | -0.022 | (-0.198, 0.153) | 0.8026 |  | -0.080 | (-0.262, 0.102) | 0.3881 |
|  |  | 16 y |  | -0.026 | (-0.208, 0.156) | 0.7784 |  | 0.009 | (-0.172, 0.189) | 0.9263 |  | -0.071 | (-0.258, 0.116) | 0.4579 |
|  |  | 17 y |  | -0.050 | (-0.229, 0.128) | 0.5800 |  | -0.034 | (-0.21, 0.142) | 0.7038 |  | -0.068 | (-0.251, 0.114) | 0.4625 |
|  |  | 18-24 y (ref) | 2068 | 0 |  |  | 2068 | 0.000 |  |  | 1726 | 0.000 |  |  |
|  | Age at 1st pregnancy | 10-14 y |  | -0.369 | (-0.82, 0.082) | 0.1091 |  | -0.407 | (-0.854, 0.039) | 0.0738 |  | -0.135 | (-0.613, 0.343) | 0.5795 |
|  |  | 15 y |  | -0.206 | (-0.422, 0.01) | 0.0611 |  | -0.196 | (-0.411, 0.018) | 0.0732 |  | -0.107 | (-0.331, 0.117) | 0.3487 |
|  |  | 16 y |  | -0.190 | (-0.368, -0.011) | 0.0373 |  | -0.169 | (-0.347, 0.01) | 0.0637 |  | -0.094 | (-0.284, 0.095) | 0.3285 |
|  |  | 17 y |  | -0.249 | (-0.402, -0.096) | 0.0015 |  | -0.242 | (-0.395, -0.09) | 0.0018 |  | -0.154 | (-0.313, 0.004) | 0.0562 |
|  |  | 18 y |  | -0.233 | (-0.409, -0.057) | 0.0094 |  | -0.220 | (-0.394, -0.046) | 0.0132 |  | -0.200 | (-0.38, -0.019) | 0.0301 |
|  |  | 19 y |  | -0.099 | (-0.257, 0.059) | 0.2204 |  | -0.075 | (-0.23, 0.081) | 0.3460 |  | -0.044 | (-0.204, 0.117) | 0.5928 |
|  |  | 20-30 y (ref) |  | 0.000 |  |  |  | 0.000 |  |  |  | 0.000 |  |  |
| Weight-for-age z score 6 to 12m | Age married | 10-13 y |  | -0.088 | (-0.334, 0.159) | 0.4855 |  | -0.015 | (-0.261, 0.231) | 0.9072 |  | -0.013 | (-0.269, 0.242) | 0.9185 |
|  |  | 14 y |  | 0.007 | (-0.17, 0.184) | 0.9400 |  | 0.061 | (-0.119, 0.241) | 0.5059 |  | 0.043 | (-0.144, 0.231) | 0.6504 |
|  |  | 15 y |  | 0.027 | (-0.14, 0.193) | 0.7528 |  | 0.047 | (-0.12, 0.214) | 0.5804 |  | 0.004 | (-0.171, 0.179) | 0.9665 |
|  |  | 16 y |  | 0.051 | (-0.12, 0.223) | 0.5587 |  | 0.043 | (-0.128, 0.214) | 0.6207 |  | -0.020 | (-0.199, 0.16) | 0.8308 |
|  |  | 17 y |  | 0.018 | (-0.151, 0.187) | 0.8370 |  | 0.002 | (-0.165, 0.168) | 0.9858 |  | 0.008 | (-0.166, 0.183) | 0.9239 |
|  |  | 18-24 y (ref) | 2048 | 0.000 |  |  | 2047 | 0.000 |  |  | 1708 | 0.000 |  |  |
|  | Age at 1st pregnancy | 10-14 y |  | -0.421 | (-0.84, -0.002) | 0.0490 |  | -0.435 | (-0.851, -0.02) | 0.0399 |  | -0.152 | (-0.6, 0.295) | 0.5048 |
|  |  | 15 y |  | -0.100 | (-0.304, 0.104) | 0.3368 |  | -0.094 | (-0.298, 0.109) | 0.3649 |  | -0.007 | (-0.221, 0.208) | 0.9506 |
|  |  | 16 y |  | -0.075 | (-0.244, 0.093) | 0.3827 |  | -0.065 | (-0.234, 0.104) | 0.4523 |  | -0.039 | (-0.22, 0.143) | 0.6768 |
|  |  | 17 y |  | -0.120 | (-0.265, 0.025) | 0.1054 |  | -0.120 | (-0.265, 0.024) | 0.1029 |  | -0.063 | (-0.215, 0.089) | 0.4158 |
|  |  | 18 y |  | -0.007 | (-0.173, 0.159) | 0.9342 |  | -0.014 | (-0.179, 0.15) | 0.8669 |  | -0.032 | (-0.205, 0.14) | 0.7161 |
|  |  | 19 y |  | -0.059 | (-0.208, 0.091) | 0.4397 |  | -0.055 | (-0.203, 0.093) | 0.4665 |  | -0.048 | (-0.201, 0.106) | 0.5415 |
|  |  | 20-30 y (ref) |  | 0.000 |  |  |  | 0.000 |  |  |  | 0.000 |  |  |
| Weight-for-length z score 6 to 12m | Age married | 10-13 y |  | 0.118 | (-0.111, 0.347) | 0.3118 |  | 0.148 | (-0.082, 0.379) | 0.2080 |  | 0.180 | (-0.068, 0.428) | 0.1543 |
|  |  | 14 y |  | 0.055 | (-0.11, 0.22) | 0.5120 |  | 0.079 | (-0.089, 0.246) | 0.3583 |  | 0.091 | (-0.09, 0.271) | 0.3253 |
|  |  | 15 y |  | 0.109 | (-0.045, 0.264) | 0.1657 |  | 0.111 | (-0.045, 0.267) | 0.1635 |  | 0.108 | (-0.061, 0.277) | 0.2097 |
|  |  | 16 y |  | 0.056 | (-0.103, 0.215) | 0.4920 |  | 0.035 | (-0.125, 0.195) | 0.6668 |  | 0.020 | (-0.153, 0.194) | 0.8182 |
|  |  | 17 y |  | 0.072 | (-0.084, 0.228) | 0.3639 |  | 0.053 | (-0.102, 0.209) | 0.5010 |  | 0.098 | (-0.071, 0.267) | 0.2561 |
|  |  | 18-24 y (ref) | 2042 | 0.000 |  |  | 2041 | 0.000 |  |  | 1703 | 0.000 |  |  |
|  | Age at 1st pregnancy | 10-14 y |  | -0.272 | (-0.665, 0.121) | 0.1744 |  | -0.271 | (-0.664, 0.122) | 0.1772 |  | -0.097 | (-0.538, 0.344) | 0.6660 |
|  |  | 15 y |  | 0.016 | (-0.173, 0.206) | 0.8643 |  | 0.023 | (-0.167, 0.213) | 0.8117 |  | 0.041 | (-0.166, 0.248) | 0.6964 |
|  |  | 16 y |  | 0.031 | (-0.126, 0.187) | 0.7015 |  | 0.033 | (-0.125, 0.19) | 0.6856 |  | -0.005 | (-0.179, 0.17) | 0.9579 |
|  |  | 17 y |  | 0.026 | (-0.109, 0.161) | 0.7049 |  | 0.025 | (-0.11, 0.161) | 0.7139 |  | 0.020 | (-0.126, 0.167) | 0.7846 |
|  |  | 18 y |  | 0.155 | (0.001, 0.309) | 0.0479 |  | 0.145 | (-0.009, 0.298) | 0.0653 |  | 0.096 | (-0.071, 0.263) | 0.2585 |
|  |  | 19 y |  | 0.005 | (-0.134, 0.144) | 0.9434 |  | 0.000 | (-0.138, 0.139) | 0.9946 |  | -0.040 | (-0.188, 0.109) | 0.6014 |
|  |  | 20-30 y (ref) |  | 0.000 |  |  |  | 0.000 |  |  |  | 0.000 |  |  |
| Head circumference-for-age z score 6 to 12m | Age married | 10-13 y |  | -0.030 | (-0.266, 0.205) | 0.8009 |  | 0.063 | (-0.172, 0.299) | 0.5979 |  | -0.008 | (-0.254, 0.238) | 0.9465 |
|  |  | 14 y |  | 0.056 | (-0.117, 0.228) | 0.5275 |  | 0.121 | (-0.053, 0.296) | 0.1716 |  | 0.107 | (-0.075, 0.29) | 0.2498 |
|  |  | 15 y |  | 0.049 | (-0.112, 0.209) | 0.5532 |  | 0.102 | (-0.059, 0.263) | 0.2133 |  | 0.050 | (-0.119, 0.218) | 0.5642 |
|  |  | 16 y |  | 0.084 | (-0.081, 0.249) | 0.3184 |  | 0.112 | (-0.053, 0.276) | 0.1823 |  | 0.025 | (-0.148, 0.198) | 0.7789 |
|  |  | 17 y |  | 0.040 | (-0.122, 0.202) | 0.6302 |  | 0.056 | (-0.104, 0.216) | 0.4942 |  | 0.081 | (-0.087, 0.25) | 0.3435 |
|  |  | 18-24 y (ref) | 2064 | 0.000 |  |  | 2064 | 0.000 |  |  | 1721 | 0.000 |  |  |
|  | Age at 1st pregnancy | 10-14 y |  | -0.367 | (-0.77, 0.036) | 0.0743 |  | -0.364 | (-0.764, 0.035) | 0.0741 |  | -0.208 | (-0.641, 0.225) | 0.3472 |
|  |  | 15 y |  | -0.104 | (-0.301, 0.093) | 0.3011 |  | -0.102 | (-0.298, 0.095) | 0.3095 |  | -0.065 | (-0.273, 0.143) | 0.5378 |
|  |  | 16 y |  | -0.144 | (-0.307, 0.019) | 0.0835 |  | -0.126 | (-0.289, 0.036) | 0.1280 |  | -0.078 | (-0.253, 0.097) | 0.3832 |
|  |  | 17 y |  | -0.120 | (-0.26, 0.02) | 0.0930 |  | -0.120 | (-0.26, 0.02) | 0.0921 |  | -0.080 | (-0.228, 0.067) | 0.2838 |
|  |  | 18 y |  | -0.090 | (-0.25, 0.07) | 0.2686 |  | -0.087 | (-0.245, 0.071) | 0.2815 |  | -0.113 | (-0.28, 0.054) | 0.1838 |
|  |  | 19 y |  | -0.059 | (-0.203, 0.085) | 0.4188 |  | -0.044 | (-0.187, 0.098) | 0.5413 |  | -0.053 | (-0.202, 0.095) | 0.4809 |
|  |  | 20-30 y (ref) |  | 0.000 |  |  |  | 0.000 |  |  |  | 0.000 |  |  |

Legend: Model 1 minimally adjusted for clustering using random effects and child age as a fixed effect. Model 2 additionally adjusted for maternal and paternal education, asset tertiles, caste, land ownership, seasonality (cosinor terms), randomization strata, study arm and child sex as fixed effects. Model 3 additionally adjusted for height of the mother. Grey shaded cells in the *p* value column indicate p<0.05.

**Table S13. Odds ratios for stunting and wasting in infants 180 to 365 days by age of marriage, age of first pregnancy and by age of marriage and pregnancy (mutually adjusted), showing models minimally adjusted, and adjusted with and without height of mother in the model. Odds ratios plotted in Figure 6.**

|  |  |  | **Model 1: Minimally adjusted (only cluster and child age)** | | | | **Model 2: Adjusted (multiple covariates without mother's height)** | | | | **Model 3: Adjusted (multiple covariates including mother's height)** | | | |
| --- | --- | --- | --- | --- | --- | --- | --- | --- | --- | --- | --- | --- | --- | --- |
| **Outcome** | **Exposure** | **Age group** | **n** | **OR** | **(95% CI)** | ***p* value** | **n** | **OR** | **(95% CI)** | ***p* value** | **n** | **OR** | **(95% CI)** | ***p value*** |
| Stunting 6 to 12m | Age married | 10-13 y |  | 1.452 | (0.907, 2.324) | 0.1200 |  | 1.223 | (0.754, 1.982) | 0.4146 |  | 1.224 | (0.71, 2.11) | 0.4660 |
|  |  | 14 y |  | 1.381 | (0.982, 1.942) | 0.0631 |  | 1.215 | (0.854, 1.727) | 0.2793 |  | 1.180 | (0.789, 1.766) | 0.4200 |
|  |  | 15 y |  | 1.252 | (0.916, 1.711) | 0.1583 |  | 1.134 | (0.822, 1.564) | 0.4448 |  | 1.191 | (0.825, 1.72) | 0.3507 |
|  |  | 16 y |  | 1.030 | (0.746, 1.424) | 0.8563 |  | 0.985 | (0.706, 1.375) | 0.9295 |  | 1.099 | (0.752, 1.604) | 0.6265 |
|  |  | 17 y |  | 1.212 | (0.868, 1.693) | 0.2589 |  | 1.207 | (0.857, 1.701) | 0.2811 |  | 1.239 | (0.839, 1.83) | 0.2810 |
|  |  | 18-24 y (ref) | 2068 | 1.000 |  |  | 2068 | 1.000 |  |  | 1726 | 1.000 |  |  |
| Wasting 6 to 12m | Age married | 10-13 y |  | 1.156 | (0.639, 2.09) | 0.6324 |  | 1.068 | (0.583, 1.956) | 0.8306 |  | 0.923 | (0.466, 1.828) | 0.8182 |
|  |  | 14 y |  | 1.045 | (0.681, 1.604) | 0.8399 |  | 1.061 | (0.684, 1.648) | 0.7908 |  | 0.955 | (0.588, 1.551) | 0.8518 |
|  |  | 15 y |  | 0.804 | (0.534, 1.21) | 0.2952 |  | 0.821 | (0.541, 1.247) | 0.3556 |  | 0.870 | (0.554, 1.367) | 0.5464 |
|  |  | 16 y |  | 0.884 | (0.585, 1.337) | 0.5591 |  | 0.919 | (0.603, 1.401) | 0.6937 |  | 0.900 | (0.566, 1.432) | 0.6576 |
|  |  | 17 y |  | 1.208 | (0.797, 1.829) | 0.3728 |  | 1.233 | (0.809, 1.878) | 0.3303 |  | 1.132 | (0.712, 1.8) | 0.5991 |
|  |  | 18-24 y (ref) | 2042 | 1.000 |  |  | 2041 | 1.000 |  |  | 1703 | 1.000 |  |  |
| Stunting 6 to 12m | Age of 1st pregnancy | 10-14 y |  | 2.623 | (1.226, 5.615) | 0.0130 |  | 2.712 | (1.233, 5.967) | 0.0131 |  | 2.122 | (0.837, 5.378) | 0.1128 |
|  |  | 15 y |  | 1.487 | (1.029, 2.151) | 0.0349 |  | 1.378 | (0.943, 2.013) | 0.0977 |  | 1.145 | (0.744, 1.763) | 0.5381 |
|  |  | 16 y |  | 1.402 | (1.033, 1.902) | 0.0302 |  | 1.336 | (0.973, 1.835) | 0.0731 |  | 1.120 | (0.775, 1.62) | 0.5454 |
|  |  | 17 y |  | 1.238 | (0.95, 1.613) | 0.1143 |  | 1.224 | (0.93, 1.611) | 0.1500 |  | 1.152 | (0.846, 1.568) | 0.3693 |
|  |  | 18 y |  | 1.267 | (0.917, 1.75) | 0.1508 |  | 1.269 | (0.909, 1.773) | 0.1617 |  | 1.198 | (0.824, 1.744) | 0.3439 |
|  |  | 19 y |  | 1.229 | (0.912, 1.658) | 0.1761 |  | 1.2 | (0.881, 1.635) | 0.2473 |  | 1.045 | (0.74, 1.476) | 0.8027 |
|  |  | 20-30 y (ref) | 2281 | 1.000 |  |  | 2265 | 1.000 |  |  | 1881 | 1.000 |  |  |
| Wasting 6 to 12m | Age of 1st pregnancy | 10-14 y |  | 2.077 | (0.875, 4.932) | 0.0976 |  | 1.71 | (0.71, 4.118) | 0.2315 |  | 1.418 | (0.486, 4.133) | 0.5225 |
|  |  | 15 y |  | 1.076 | (0.673, 1.722) | 0.7596 |  | 0.992 | (0.616, 1.598) | 0.9746 |  | 1.017 | (0.597, 1.73) | 0.9513 |
|  |  | 16 y |  | 0.971 | (0.657, 1.434) | 0.8807 |  | 0.896 | (0.602, 1.334) | 0.5895 |  | 0.993 | (0.634, 1.554) | 0.9747 |
|  |  | 17 y |  | 0.960 | (0.688, 1.34) | 0.8119 |  | 0.911 | (0.649, 1.279) | 0.5911 |  | 0.976 | (0.668, 1.425) | 0.8993 |
|  |  | 18 y |  | 0.654 | (0.418, 1.024) | 0.0636 |  | 0.628 | (0.398, 0.989) | 0.0447 |  | 0.731 | (0.446, 1.198) | 0.2143 |
|  |  | 19 y |  | 0.920 | (0.63, 1.344) | 0.6657 |  | 0.871 | (0.593, 1.279) | 0.4811 |  | 0.937 | (0.614, 1.429) | 0.7618 |
|  |  | 20-30 y (ref) | 2252 | 1.000 |  |  | 2235 | 1.000 |  |  | 1855 | 1.000 |  |  |
| Stunting 6 to 12m | Age married | 10-13 y |  | 1.135 | (0.684, 1.883) | 0.6248 |  | 0.929 | (0.55, 1.57) | 0.7831 |  | 1.040 | (0.582, 1.857) | 0.8949 |
|  |  | 14 y |  | 1.174 | (0.81, 1.701) | 0.3974 |  | 1.017 | (0.689, 1.501) | 0.9326 |  | 1.069 | (0.688, 1.662) | 0.7661 |
|  |  | 15 y |  | 1.054 | (0.742, 1.497) | 0.7689 |  | 0.958 | (0.666, 1.379) | 0.8184 |  | 1.092 | (0.725, 1.644) | 0.6743 |
|  |  | 16 y |  | 0.871 | (0.604, 1.257) | 0.4611 |  | 0.838 | (0.573, 1.224) | 0.3599 |  | 0.993 | (0.648, 1.52) | 0.9737 |
|  |  | 17 y |  | 1.078 | (0.755, 1.54) | 0.6780 |  | 1.081 | (0.749, 1.56) | 0.6770 |  | 1.148 | (0.759, 1.738) | 0.5134 |
|  |  | 18-24 y (ref) | 2068 | 1.000 |  |  | 2068 | 1.000 |  |  | 1726 | 1.000 |  |  |
|  | Age at 1st pregnancy | 10-14 y |  | 2.832 | (1.232, 6.508) | 0.0142 |  | 3.276 | (1.379, 7.784) | 0.0072 |  | 2.387 | (0.882, 6.463) | 0.0868 |
|  |  | 15 y |  | 1.484 | (0.977, 2.255) | 0.0644 |  | 1.461 | (0.946, 2.254) | 0.0871 |  | 1.151 | (0.704, 1.88) | 0.5751 |
|  |  | 16 y |  | 1.497 | (1.054, 2.126) | 0.0243 |  | 1.457 | (1.011, 2.1) | 0.0434 |  | 1.220 | (0.802, 1.856) | 0.3519 |
|  |  | 17 y |  | 1.377 | (1.015, 1.868) | 0.0400 |  | 1.367 | (0.997, 1.874) | 0.0521 |  | 1.194 | (0.84, 1.699) | 0.3232 |
|  |  | 18 y |  | 1.367 | (0.965, 1.937) | 0.0783 |  | 1.335 | (0.931, 1.914) | 0.1159 |  | 1.242 | (0.83, 1.859) | 0.2911 |
|  |  | 19 y |  | 1.282 | (0.936, 1.756) | 0.1214 |  | 1.25 | (0.905, 1.727) | 0.1759 |  | 1.090 | (0.76, 1.564) | 0.6398 |
|  |  | 20-30 y (ref) |  | 1.000 |  |  |  | 1.000 |  |  |  | 1.000 |  |  |
| Wasting 6 to 12m | Age married | 10-13 y |  | 0.922 | (0.482, 1.763) | 0.8052 |  | 0.898 | (0.462, 1.744) | 0.7509 |  | 0.801 | (0.384, 1.67) | 0.5545 |
|  |  | 14 y |  | 0.904 | (0.562, 1.454) | 0.6780 |  | 0.956 | (0.584, 1.565) | 0.8571 |  | 0.855 | (0.498, 1.469) | 0.5704 |
|  |  | 15 y |  | 0.737 | (0.464, 1.172) | 0.1975 |  | 0.791 | (0.492, 1.27) | 0.3311 |  | 0.800 | (0.481, 1.333) | 0.3927 |
|  |  | 16 y |  | 0.854 | (0.534, 1.367) | 0.5109 |  | 0.925 | (0.572, 1.497) | 0.7514 |  | 0.845 | (0.499, 1.432) | 0.5322 |
|  |  | 17 y |  | 1.245 | (0.798, 1.943) | 0.3340 |  | 1.303 | (0.829, 2.048) | 0.2518 |  | 1.119 | (0.682, 1.836) | 0.6577 |
|  |  | 18-24 y (ref) | 2042 | 1.000 |  |  | 2041 | 1.000 |  |  | 1703 | 1.000 |  |  |
|  | Age at 1st pregnancy | 10-14 y |  | 2.552 | (0.995, 6.546) | 0.0513 |  | 2.128 | (0.81, 5.59) | 0.1253 |  | 1.861 | (0.591, 5.859) | 0.2882 |
|  |  | 15 y |  | 1.375 | (0.801, 2.359) | 0.2483 |  | 1.255 | (0.721, 2.185) | 0.4224 |  | 1.265 | (0.68, 2.353) | 0.4582 |
|  |  | 16 y |  | 1.099 | (0.692, 1.744) | 0.6892 |  | 0.989 | (0.615, 1.591) | 0.9649 |  | 1.167 | (0.686, 1.984) | 0.5682 |
|  |  | 17 y |  | 1.042 | (0.706, 1.537) | 0.8377 |  | 0.967 | (0.649, 1.441) | 0.8676 |  | 1.123 | (0.721, 1.75) | 0.6079 |
|  |  | 18 y |  | 0.728 | (0.453, 1.171) | 0.1909 |  | 0.707 | (0.435, 1.149) | 0.1619 |  | 0.882 | (0.521, 1.495) | 0.6419 |
|  |  | 19 y |  | 1.016 | (0.684, 1.509) | 0.9371 |  | 0.958 | (0.64, 1.434) | 0.8341 |  | 1.092 | (0.701, 1.701) | 0.6965 |
|  |  | 20-30 y (ref) |  | 1.000 |  |  |  | 1.000 |  |  |  | 1.000 |  |  |

Legend: Model 1 minimally adjusted for clustering using random effects and child age as a fixed effect. Model 2 additionally adjusted for maternal and paternal education, asset tertiles, caste, land ownership, seasonality (cosinor terms), randomization strata, study arm and child sex as fixed effects. Model 3 additionally adjusted for height of the mother. Grey shaded cells in the *p* value column indicate p<0.05.

**Table S14.** **Coefficients from mixed linear regressions of neonatal anthropometric z scores in children of mothers married <16 years of age by age at marriage and age at first pregnancy for length-, weight- and head circumference-for-age, and weight-for-length z scores at <8 days, showing models minimally adjusted, and adjusted without height of mother in the model.** Coefficients for minimally adjusted and fully adjusted without maternal height plotted in Figure S3**.**

|  |  |  | Model 1: Minimally adjusted (only cluster) | | | | Model 2: Adjusted (multiple covariates without mother's height) | | | |
| --- | --- | --- | --- | --- | --- | --- | --- | --- | --- | --- |
| Outcome | Exposure | Age group | n | Coeff. | (95% CI) | *p* value | n | Coeff. | (95% CI) | *p* value |
| Length-for-age z score <8 days in children of women married <16 years of age | Age married | 10-13 y |  | -0.083 | (-0.361, 0.196) | 0.5610 |  | -0.045 | (-0.321, 0.231) | 0.7482 |
|  |  | 14-15 y (ref) | 613 | 0.000 |  |  | 613 | 0.000 |  |  |
|  | Age at 1st pregnancy | 10-14 y |  | -0.111 | (-0.675, 0.453) | 0.6996 |  | -0.333 | (-0.901, 0.235) | 0.2506 |
|  |  | 15 y |  | -0.281 | (-0.604, 0.041) | 0.0876 |  | -0.455 | (-0.782, -0.129) | 0.0062 |
|  |  | 16 y |  | -0.248 | (-0.543, 0.046) | 0.0987 |  | -0.432 | (-0.728, -0.135) | 0.0043 |
|  |  | 17 y |  | -0.135 | (-0.433, 0.164) | 0.3773 |  | -0.319 | (-0.62, -0.019) | 0.0370 |
|  |  | 18 y |  | -0.359 | (-0.77, 0.051) | 0.0862 |  | -0.466 | (-0.869, -0.064) | 0.0232 |
|  |  | 19 y |  | -0.104 | (-0.464, 0.256) | 0.5709 |  | -0.216 | (-0.571, 0.139) | 0.2326 |
|  |  | 20-30 y (ref) |  | 0.000 |  |  |  | 0.000 |  |  |
| Weight-for-age z score <8 days in children of women married <16 years of age | Age married | 10-13 y |  | 0.046 | (-0.203, 0.296) | 0.7151 |  | 0.133 | (-0.113, 0.379) | 0.2903 |
|  |  | 14-15 y (ref) | 620 | 0.000 |  |  | 620 | 0.000 |  |  |
|  | Age at 1st pregnancy | 10-14 y |  | -0.503 | (-1.008, 0.001) | 0.0504 |  | -0.774 | (-1.278, -0.271) | 0.0026 |
|  |  | 15 y |  | -0.454 | (-0.74, -0.167) | 0.0019 |  | -0.657 | (-0.943, -0.372) | 0.0000 |
|  |  | 16 y |  | -0.484 | (-0.746, -0.221) | 0.0003 |  | -0.642 | (-0.903, -0.382) | 0.0000 |
|  |  | 17 y |  | -0.431 | (-0.698, -0.165) | 0.0015 |  | -0.540 | (-0.805, -0.276) | 0.0001 |
|  |  | 18 y |  | -0.421 | (-0.788, -0.053) | 0.0247 |  | -0.492 | (-0.849, -0.134) | 0.0070 |
|  |  | 19 y |  | -0.160 | (-0.483, 0.162) | 0.3299 |  | -0.273 | (-0.588, 0.042) | 0.0898 |
|  |  | 20-30 y (ref) |  | 0.000 |  |  |  | 0.000 |  |  |
| Weight-for-length z score <8 days in children of women married <16 years of age | Age married | 10-13 y |  | 0.047 | (-0.292, 0.386) | 0.7845 |  | 0.180 | (-0.148, 0.507) | 0.2816 |
|  |  | 14-15 y (ref) | 523 | 0.000 |  |  | 523 | 0.000 |  |  |
|  | Age at 1st pregnancy | 10-14 y |  | -0.617 | (-1.29, 0.056) | 0.0725 |  | -0.958 | (-1.613, -0.304) | 0.0041 |
|  |  | 15 y |  | -0.381 | (-0.761, -0.001) | 0.0492 |  | -0.627 | (-0.994, -0.26) | 0.0008 |
|  |  | 16 y |  | -0.384 | (-0.741, -0.028) | 0.0346 |  | -0.497 | (-0.84, -0.153) | 0.0046 |
|  |  | 17 y |  | -0.470 | (-0.819, -0.12) | 0.0084 |  | -0.522 | (-0.857, -0.188) | 0.0022 |
|  |  | 18 y |  | -0.205 | (-0.703, 0.294) | 0.4207 |  | -0.256 | (-0.729, 0.216) | 0.2873 |
|  |  | 19 y |  | -0.284 | (-0.717, 0.148) | 0.1977 |  | -0.385 | (-0.796, 0.027) | 0.0669 |
|  |  | 20-30 y (ref) |  | 0.000 |  |  |  | 0.000 |  |  |
| Head circumference-for-age z score <8 days in children of women married <16 years of age | Age married | 10-13 y |  | 0.030 | (-0.266, 0.326) | 0.8439 |  | 0.079 | (-0.216, 0.373) | 0.5998 |
|  |  | 14-15 y (ref) | 612 | 0.000 |  |  | 612 | 0.000 |  |  |
|  | Age at 1st pregnancy | 10-14 y |  | -0.602 | (-1.196, -0.008) | 0.0469 |  | -0.777 | (-1.376, -0.179) | 0.0110 |
|  |  | 15 y |  | -0.203 | (-0.543, 0.136) | 0.2407 |  | -0.362 | (-0.704, -0.019) | 0.0385 |
|  |  | 16 y |  | -0.376 | (-0.688, -0.065) | 0.0179 |  | -0.513 | (-0.826, -0.2) | 0.0013 |
|  |  | 17 y |  | -0.280 | (-0.595, 0.034) | 0.0809 |  | -0.385 | (-0.701, -0.068) | 0.0172 |
|  |  | 18 y |  | -0.343 | (-0.775, 0.09) | 0.1203 |  | -0.418 | (-0.843, 0.007) | 0.0538 |
|  |  | 19 y |  | -0.021 | (-0.4, 0.358) | 0.9117 |  | -0.127 | (-0.501, 0.248) | 0.5081 |
|  |  | 20-30 y (ref) |  | 0.000 |  |  |  | 0.000 |  |  |

Legend: Model 1 minimally adjusted for clustering using random effects in all models. Model 2 additionally adjusted for maternal and paternal education, asset tertiles, caste, land ownership, seasonality (cosinor terms), randomization strata, study arm and child sex as fixed effects. Grey shaded cells in the *p* value column indicate p<0.05.

**Table S15.** **Odds ratios from mixed logistic regressions of neonatal and infant stunting and wasting in children of mothers married <16 years of age by age at marriage and age at first pregnancy for length-, weight- and head circumference-for-age, and weight-for-length z scores at <8 days and from 180 to 365 days, showing models minimally adjusted, and adjusted without height of mother in the model.** Coefficients for minimally adjusted and fully adjusted without maternal height plotted in Figure S5**.**

|  |  |  | Model 1: Minimally adjusted in children of mothers married <16 years | | | | Model 2: Adjusted (multiple covariates not including mother's height) in children of mothers married <16 years | | | |
| --- | --- | --- | --- | --- | --- | --- | --- | --- | --- | --- |
| Outcome | Exposure | Age group | n | OR | (95% CI) | p value | n | OR | (95% CI) | p value |
| Stunting<8 days in children of women married <16 years of age | Age married | 10-13 y |  | 1.171 | (0.66, 2.079) | 0.5889 |  | 1.153 | (0.637, 2.089) | 0.6378 |
|  |  | 14-15 y (ref) | 613 | 1.000 |  |  | 613 | 1.000 |  |  |
|  | Age at 1st pregnancy | 10-14 y |  | 1.056 | (0.329, 3.387) | 0.9266 |  | 1.420 | (0.417, 4.838) | 0.5750 |
|  |  | 15 y |  | 1.216 | (0.622, 2.377) | 0.5672 |  | 1.622 | (0.798, 3.297) | 0.1816 |
|  |  | 16 y |  | 1.356 | (0.737, 2.496) | 0.3275 |  | 1.764 | (0.925, 3.362) | 0.0847 |
|  |  | 17 y |  | 0.766 | (0.4, 1.465) | 0.4197 |  | 0.970 | (0.491, 1.916) | 0.9308 |
|  |  | 18 y |  | 1.535 | (0.669, 3.522) | 0.3125 |  | 1.905 | (0.814, 4.457) | 0.1374 |
|  |  | 19 y |  | 1.240 | (0.589, 2.609) | 0.5712 |  | 1.469 | (0.685, 3.151) | 0.3228 |
|  |  | 20-30 y (ref) |  | 1.000 |  |  |  | 1.000 |  |  |
| Wasting<8 days in children of women married <16 years of age | Age married | 10-13 y |  | 1.299 | (0.634, 2.665) | 0.4748 |  | 1.188 | (0.546, 2.588) | 0.6639 |
|  |  | 14-15 y (ref) | 523 | 1.000 |  |  | 523 | 1.000 |  |  |
|  | Age at 1st pregnancy | 10-14 y |  | 0.941 | (0.215, 4.121) | 0.9358 |  | 1.250 | (0.257, 6.088) | 0.7820 |
|  |  | 15 y |  | 0.912 | (0.381, 2.184) | 0.8361 |  | 1.114 | (0.437, 2.844) | 0.8209 |
|  |  | 16 y |  | 1.001 | (0.444, 2.261) | 0.9972 |  | 1.164 | (0.487, 2.781) | 0.7333 |
|  |  | 17 y |  | 1.421 | (0.655, 3.085) | 0.3738 |  | 1.366 | (0.601, 3.105) | 0.4569 |
|  |  | 18 y |  | 0.947 | (0.294, 3.045) | 0.9267 |  | 0.768 | (0.215, 2.744) | 0.6846 |
|  |  | 19 y |  | 0.839 | (0.3, 2.344) | 0.7374 |  | 0.765 | (0.256, 2.29) | 0.6320 |
|  |  | 20-30 y (ref) |  | 1.000 |  |  |  | 1.000 |  |  |
| Stunting 6 to 12m in children of women married <16 years of age | Age married | 10-13 y |  | 1.038 | (0.664, 1.622) | 0.8706 |  | 0.966 | (0.606, 1.541) | 0.8858 |
|  |  | 14-15 y (ref) | 942 | 1.000 |  |  | 942 | 1.000 |  |  |
|  | Age at 1st pregnancy | 10-14 y |  | 2.767 | (1.183, 6.47) | 0.0189 |  | 3.012 | (1.23, 7.381) | 0.0159 |
|  |  | 15 y |  | 1.438 | (0.906, 2.281) | 0.1231 |  | 1.313 | (0.808, 2.135) | 0.2716 |
|  |  | 16 y |  | 1.390 | (0.902, 2.142) | 0.1354 |  | 1.325 | (0.835, 2.102) | 0.2319 |
|  |  | 17 y |  | 1.434 | (0.924, 2.225) | 0.1081 |  | 1.342 | (0.848, 2.124) | 0.2086 |
|  |  | 18 y |  | 1.035 | (0.572, 1.874) | 0.9092 |  | 0.912 | (0.493, 1.685) | 0.7677 |
|  |  | 19 y |  | 1.224 | (0.73, 2.053) | 0.4425 |  | 1.126 | (0.656, 1.931) | 0.6671 |
|  |  | 20-30 y (ref) |  | 1.000 |  |  |  | 1.000 |  |  |
| Wasting 6 to 12m in children of women married <16 years of age | Age married | 10-13 y |  | 1.113 | (0.618, 2.004) | 0.7206 |  | 0.970 | (0.532, 1.769) | 0.9215 |
|  |  | 14-15 y (ref) | 928 | 1.000 |  |  | 928 | 1.000 |  |  |
|  | Age at 1st pregnancy | 10-14 y |  | 2.693 | (0.993, 7.3) | 0.0516 |  | 2.239 | (0.794, 6.314) | 0.1275 |
|  |  | 15 y |  | 1.447 | (0.775, 2.699) | 0.2458 |  | 1.426 | (0.747, 2.724) | 0.2824 |
|  |  | 16 y |  | 1.057 | (0.581, 1.925) | 0.8552 |  | 1.027 | (0.549, 1.924) | 0.9327 |
|  |  | 17 y |  | 0.895 | (0.475, 1.689) | 0.7326 |  | 0.842 | (0.438, 1.618) | 0.6056 |
|  |  | 18 y |  | 1.208 | (0.558, 2.615) | 0.6314 |  | 1.060 | (0.481, 2.333) | 0.8853 |
|  |  | 19 y |  | 0.986 | (0.478, 2.035) | 0.9693 |  | 0.872 | (0.412, 1.848) | 0.7210 |
|  |  | 20-30 y (ref) |  | 1.000 |  |  |  | 1.000 |  |  |

Legend: Model 1 minimally adjusted for clustering using random effects in all models and child age as a fixed effect in infant models (180-365 days). Model 2 additionally adjusted for maternal and paternal education, asset tertiles, caste, land ownership, seasonality (cosinor terms), randomization strata, study arm and child sex as fixed effects. Grey shaded cells in the *p* value column indicate p<0.05.

**Table S16.** **Coefficients from mixed linear regressions of infant anthropometric z scores in children of mothers married <16 years of age by age at marriage and age at first pregnancy for length-, weight- and head circumference-for-age, and weight-for-length z scores from 180 to 365 days, showing models minimally adjusted, and adjusted without height of mother in the model.** Coefficients for minimally adjusted and fully adjusted without maternal height plotted in Figure S4**.**

|  |  |  | Model 1: Minimally adjusted (only cluster) | | | | Model 2: Adjusted (multiple covariates without mother's height) | | | |
| --- | --- | --- | --- | --- | --- | --- | --- | --- | --- | --- |
| Outcome | Exposure | Age group | n | Coeff. | (95% CI) | *p* value | n | Coeff. | (95% CI) | *p* value |
| Length-for-age z score at 6-12m in children of women married <16 years of age | Age married | 10-13 y |  | -0.210 | (-0.454, 0.035) | 0.0927 |  | -0.162 | (-0.406, 0.083) | 0.1947 |
|  |  | 14-15 y (ref) | 942 | 0.000 |  |  | 942 | 0.000 |  |  |
|  | Age at 1st pregnancy | 10-14 y |  | -0.384 | (-0.866, 0.098) | 0.1186 |  | -0.412 | (-0.894, 0.07) | 0.0938 |
|  |  | 15 y |  | -0.229 | (-0.477, 0.019) | 0.0703 |  | -0.198 | (-0.45, 0.053) | 0.1225 |
|  |  | 16 y |  | -0.215 | (-0.447, 0.017) | 0.0689 |  | -0.194 | (-0.432, 0.043) | 0.1086 |
|  |  | 17 y |  | -0.268 | (-0.504, -0.032) | 0.0261 |  | -0.233 | (-0.47, 0.004) | 0.0541 |
|  |  | 18 y |  | -0.145 | (-0.456, 0.166) | 0.3596 |  | -0.082 | (-0.391, 0.227) | 0.6010 |
|  |  | 19 y |  | -0.260 | (-0.536, 0.016) | 0.065 |  | -0.215 | (-0.49, 0.061) | 0.1274 |
|  |  | 20-30 y (ref) |  | 0.000 |  |  |  | 0.000 |  |  |
| Weight-for-age z score at 6-12m in children of women married <16 years of age | Age married | 10-13 y |  | -0.099 | (-0.324, 0.126) | 0.3887 |  | -0.061 | (-0.286, 0.164) | 0.5963 |
|  |  | 14-15 y (ref) | 932 | 0.000 |  |  | 932 | 0.000 |  |  |
|  | Age at 1st pregnancy | 10-14 y |  | -0.444 | (-0.88, -0.008) | 0.0459 |  | -0.455 | (-0.89, -0.021) | 0.0401 |
|  |  | 15 y |  | -0.121 | (-0.352, 0.11) | 0.3042 |  | -0.123 | (-0.354, 0.109) | 0.2994 |
|  |  | 16 y |  | -0.114 | (-0.33, 0.102) | 0.3006 |  | -0.132 | (-0.35, 0.087) | 0.2369 |
|  |  | 17 y |  | -0.071 | (-0.292, 0.15) | 0.5288 |  | -0.062 | (-0.281, 0.157) | 0.5786 |
|  |  | 18 y |  | -0.086 | (-0.373, 0.202) | 0.5593 |  | -0.026 | (-0.31, 0.258) | 0.8592 |
|  |  | 19 y |  | -0.151 | (-0.406, 0.104) | 0.2446 |  | -0.136 | (-0.389, 0.117) | 0.2914 |
|  |  | 20-30 y (ref) |  | 0.000 |  |  |  | 0.000 |  |  |
| Weight-for-length z score at 6-12m in children of women married <16 years of age | Age married | 10-13 y |  | 0.040 | (-0.171, 0.251) | 0.7105 |  | 0.055 | (-0.157, 0.267) | 0.6125 |
|  |  | 14-15 y (ref) | 928 | 0.000 |  |  | 928 | 0.000 |  |  |
|  | Age at 1st pregnancy | 10-14 y |  | -0.284 | (-0.696, 0.128) | 0.1765 |  | -0.285 | (-0.698, 0.128) | 0.1762 |
|  |  | 15 y |  | -0.007 | (-0.224, 0.211) | 0.9521 |  | -0.016 | (-0.234, 0.202) | 0.8852 |
|  |  | 16 y |  | 0.007 | (-0.197, 0.21) | 0.9484 |  | -0.018 | (-0.224, 0.187) | 0.8599 |
|  |  | 17 y |  | 0.087 | (-0.121, 0.296) | 0.4118 |  | 0.085 | (-0.121, 0.292) | 0.4176 |
|  |  | 18 y |  | -0.039 | (-0.308, 0.229) | 0.7751 |  | 0.003 | (-0.263, 0.269) | 0.9821 |
|  |  | 19 y |  | 0.004 | (-0.236, 0.243) | 0.9768 |  | 0.002 | (-0.236, 0.241) | 0.9865 |
|  |  | 20-30 y (ref) |  | 0.000 |  |  |  | 0.000 |  |  |
| Head circumference-for-age z score at 6-12m in children of women married <16 years of age | Age married | 10-13 y |  | -0.089 | (-0.295, 0.118) | 0.3994 |  | -0.043 | (-0.25, 0.164) | 0.6833 |
|  |  | 14-15 y (ref) | 939 | 0.000 |  |  | 939 | 0.000 |  |  |
|  | Age at 1st pregnancy | 10-14 y |  | -0.461 | (-0.865, -0.058) | 0.0251 |  | -0.422 | (-0.826, -0.018) | 0.0404 |
|  |  | 15 y |  | -0.215 | (-0.429, -0.002) | 0.0484 |  | -0.193 | (-0.408, 0.022) | 0.0783 |
|  |  | 16 y |  | -0.307 | (-0.507, -0.107) | 0.0026 |  | -0.268 | (-0.47, -0.066) | 0.0094 |
|  |  | 17 y |  | -0.267 | (-0.471, -0.063) | 0.0104 |  | -0.249 | (-0.452, -0.046) | 0.0164 |
|  |  | 18 y |  | -0.124 | (-0.388, 0.14) | 0.3577 |  | -0.085 | (-0.347, 0.177) | 0.5246 |
|  |  | 19 y |  | -0.269 | (-0.505, -0.033) | 0.0256 |  | -0.215 | (-0.45, 0.019) | 0.0722 |
|  |  | 20-30 y (ref) |  | 0.000 |  |  |  | 0.000 |  |  |

Legend: Model 1 minimally adjusted for clustering using random effects and child age as a fixed effect. Model 2 additionally adjusted for maternal and paternal education, asset tertiles, caste, land ownership, seasonality (cosinor terms), randomization strata, study arm and child sex as fixed effects. Grey shaded cells in the *p* value column indicate p<0.05.
